# A roadmap of constitutive NF-κB activity in Hodgkin lymphoma: Dominant roles of p50 and p52 revealed by genome-wide analyses

**DOI:** 10.1186/s13073-016-0280-5

**Published:** 2016-03-17

**Authors:** Kivia A. P. de Oliveira, Eva Kaergel, Matthias Heinig, Jean-Fred Fontaine, Giannino Patone, Enrique M. Muro, Stephan Mathas, Michael Hummel, Miguel A. Andrade-Navarro, Norbert Hübner, Claus Scheidereit

**Affiliations:** Signal Transduction in Tumor Cells, Max Delbrück Center for Molecular Medicine, Robert-Rössle-Strasse 10, 13125 Berlin, Germany; Department of Computational Biology, Max Planck Institute for Molecular Genetics, 14195 Berlin, Germany; Genetics and Genomics of Cardiovascular Diseases, Max Delbrück Center for Molecular Medicine, Robert-Rössle-Strasse 10, 13125 Berlin, Germany; Institute of Computational Biology, Helmholtz Zentrum München, Ingolstädter Landstr.1, 85764 Neuherberg, Germany; Computational Biology and Data Mining, Max Delbrück Center for Molecular Medicine, Robert-Rössle-Strasse 10, 13125 Berlin, Germany; Present address: Johannes Gutenberg University, 55128 Mainz, Germany; Max-Delbrück-Center for Molecular Medicine, 13125 Berlin, Germany; Hematology, Oncology and Tumor Immunology, Charité-Universitätsmedizin Berlin, 13353 Berlin, Germany; Institute of Pathology, Charité-Universitätsmedizin Berlin, 10117 Berlin, Germany

**Keywords:** Transcription factor, ChIP sequencing, lymphoma, gene expression, promoter, enhancer, consensus sequence, B lymphocytes, cell death, inflammation

## Abstract

**Background:**

NF-κB is widely involved in lymphoid malignancies; however, the functional roles and specific transcriptomes of NF-κB dimers with distinct subunit compositions have been unclear.

**Methods:**

Using combined ChIP-sequencing and microarray analyses, we determined the cistromes and target gene signatures of canonical and non-canonical NF-κB species in Hodgkin lymphoma (HL) cells.

**Results:**

We found that the various NF-κB subunits are recruited to regions with redundant κB motifs in a large number of genes. Yet canonical and non-canonical NF-κB dimers up- and downregulate gene sets that are both distinct and overlapping, and are associated with diverse biological functions. p50 and p52 are formed through NIK-dependent p105 and p100 precursor processing in HL cells and are the predominant DNA binding subunits. Logistic regression analyses of combinations of the p50, p52, RelA, and RelB subunits in binding regions that have been assigned to genes they regulate reveal a cross-contribution of p52 and p50 to canonical and non-canonical transcriptomes. These analyses also indicate that the subunit occupancy pattern of NF-κB binding regions and their distance from the genes they regulate are determinants of gene activation versus repression. The pathway-specific signatures of activated and repressed genes distinguish HL from other NF-κB-associated lymphoid malignancies and inversely correlate with gene expression patterns in normal germinal center B cells, which are presumed to be the precursors of HL cells.

**Conclusions:**

We provide insights that are relevant for lymphomas with constitutive NF-κB activation and generally for the decoding of the mechanisms of differential gene regulation through canonical and non-canonical NF-κB signaling.

**Electronic supplementary material:**

The online version of this article (doi:10.1186/s13073-016-0280-5) contains supplementary material, which is available to authorized users.

## Background

The NF-κB family of transcription factors (TFs) includes RelA (p65), RelB, c-Rel, and the precursor proteins p105 and p100, which are processed to p50 and p52, respectively. These subunits can form various homo- and heterodimers which regulate gene expression by binding to κB sites in the promoters and enhancers of genes in a wide range of mammalian cell types. NF-κB activation can be achieved through the canonical and non-canonical signaling pathways. The canonical pathway involves the degradation of small IκB proteins and the nuclear translocation of p50-RelA. The non-canonical pathway depends on the activation of NF-κB-inducing kinase (NIK) and IκB kinase α (IKKα), resulting in the induction of NF-κB dimers containing p52 and/or RelB [1–3]. Our recent work, however, has shown that non-canonical signaling is more complex. Stimulation through LTβR results in the parallel production of p50 and p52, and both are important to assure the full range of signal-induced gene expression [4].

Activated NF-κB plays a major role in cell cycle progression and survival, immune responses, and development [5]. To control such a broad range of physiological processes, the NF-κB response relies on diverse and complex mechanisms that guarantee selectivity by limiting the activation of only subsets of potential NF-κB target genes. The regulation of selected sets of genes by distinct NF-κB subunits may depend on chromatin accessibility, differential expression, dimer formation, differential DNA recognition, and transactivation mechanisms [6]. The diverse phenotypes of mice that lack single NF-κB subunits imply that different dimers regulate distinct sets of genes [7]. However, while previous studies have mainly focused on RelA and c-Rel and revealed considerable redundancy in gene expression regulation [8], little is known about the dependence of the expression of specific genes on canonical versus non-canonical NF-κB dimers. Previous chromatin immunoprecipitation (ChIP)-based genome-wide NF-κB analyses have mostly investigated canonical NF-κB and been focused on RelA [9–11], or analyzed NF-κB subunits in B cells expressing LMP1 [12]. Nonetheless, to our knowledge, the relation between NF-κB cistromes and subunit-dependent gene regulation has not yet been explored in systematic global analyses.

Understanding how particular subunit combinations control target gene expression is especially important for cancer biology, where the development and maintenance of various solid tumors and lymphoid malignancies are driven by NF-κB [13, 14]. Within B-lymphoid malignancies, constitutive NF-κB activation has been identified in classical Hodgkin lymphoma (HL) and - to various degrees - in non-Hodgkin lymphomas including diffuse large B-cell lymphoma, mucosa-associated lymphoid tissue lymphoma, primary effusion lymphoma, adult T-cell lymphoma/leukemia, and multiple myeloma [13, 15]. The deregulation of NF-κB in these malignancies is caused by diverse mechanisms. Its activation in HL is thought to be associated with multiple genetic alterations and deregulated autocrine and/or paracrine signaling, leading to constitutive IKK activity [16]. The presence of activated RelA and of NIK in primary HL biopsies as well as in HL cell lines [17, 18] indicates a parallel constitutive activation of canonical and non-canonical IKK signaling. This makes HL an ideal model system to study the mechanisms of differential gene regulation by these two pathways.

Here we dissected the composition of activated NF-κB subunits, their cistromes, and p50/RelA- and p52/RelB-specific transcriptomes and functions in HL cells. Our results revealed that NIK promotes the formation of both p50 and p52, which account for most NF-κB activity in HL cells. Major gene expression patterns and cell survival were dependent on the non-canonical pathway. However, each of the pathways regulated both unique and overlapping transcriptomes, and these signatures were consistent with the differential gene expression patterns seen in HL biopsies. A regression analysis of binding patterns and regulation indicates a cross-contribution of p50 and p52 to canonical and non-canonical NF-κB pathways. Our study provides the basis for understanding how canonical and non-canonical NF-κB pathways regulate gene expression in tumor cells.

## Methods

### Cell lines

HL cell lines L428 (ACC 197), L1236 (ACC 530), KM-H2 (ACC 8), L591 (ACC 602), L540 (ACC 72), and HDLM-2 (ACC 17) were purchased from DSMZ (www.dsmz.de). All cell lines were cultivated in RPMI medium 1640 (Gibco) supplemented with inactivated FBS, according to the instructions of the provider.

### Immunohistochemistry

Immunohistochemical detections of p105/p50 and p100/p52 were performed as described [19]. A total of 20 biopsies were used, from which 20 samples were stained for p105/p50 (#3035, Cell Signaling), and 18 were stained for p100/p52 (HPA008422, Sigma).

### Analysis of ChIP-seq data

Short reads from the Illumina sequencer were aligned using Bowtie, retaining only uniquely mapping reads. Quality control was performed according to the ENCODE guidelines [20]. All our samples passed the stringent QC thresholds (see ‘Quality control of ChIP-seq libraries’ in Additional file 1: Supplemental Experimental Procedures). Duplicated reads that are likely to be PCR artefacts were removed using samtools. In order to be able to compare sequencing libraries with different total read counts we used random sampling to reduce all libraries to a size of 10 million reads. The genome wide distribution of binding regions was determined using MACS [21]. All ChIP-seq experiments were conducted in two biological replicates, so we investigated the reproducibility using the IDR method. After IDR analysis, reads from biological replicates were merged and jointly analyzed with MACS. Final peak calls were defined as regions with IDR <0.05 and MACS false discovery rate (FDR) <0.05. See also Additional file 1: Supplemental Experimental Procedures for details about peak calling and combinatorial analysis of NF-κB subunits.

### Analysis of gene expression data

Experiments for the knockdown of the two subunit combinations (RelA/p50 and RelB/p52) were performed and analyzed independently. Each experiment was performed in three biological replicates. For Experiment #1, each biological replicate was composed of four samples (sample 1 = siControl #1; sample 2 = siControl#2; sample 3 = siRELA #1 + siNFKB1 #1; sample 4 = siRELA #2 + siNFKB1 #2). For Experiment #2, each biological replicate was composed of an additional four samples (sample 5 = siControl #1; sample 6 = siControl#2; sample 7 = siRELB #1 + siNFKB2 #1; sample 8 = siRELB #2 + siNFKB2 #2). For the data analysis, all 24 samples were separated into four groups (Group1 = all replicates from samples 1 and 2, Group 2 = all replicates from samples 3 and 4, Group 3 = all replicates from samples 5 and 6, and Group 4 = all replicates from samples 7 and 8). Differential expression was determined using a two-sample t-test and *P* values adjusted for multiple testing using the Benjamini–Hochberg method. A gene was called differentially expressed if at least one transcript cluster assigned to the gene was called differential (FDR <0.05) and showed at least 10 % expression difference between Groups 1 and 2 or 3 and 4. See Additional file 1: Supplemental Experimental Procedures for details.

### Integration of target genes with gene expression in human lymphomas

Human lymphoma gene expression data were obtained from GEO (GSE12453). Genes with low average expression (Affymetrix hybridization signal <6) were removed. Differential expression was determined between HL and all remaining samples using the same method as described above. For details see Additional file 1.

## Results

### Predominance of p50 and p52 and non-canonical IKK signaling in the constitutive NF-κB activity in HL

**Fig. 1:**
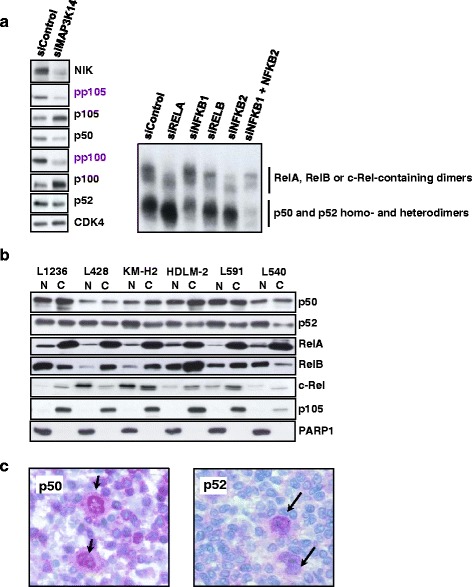
Dominant contribution of p50 and p52 in the constitutive NF-κB activity in HL cells. **a** Left: RNAi-mediated knockdown (KD) of NIK (*MAP3K14*). L1236 cells were incubated with siRNA against *MAP3K14* and harvested 1 day after the end of the siRNA treatment. Protein levels of the precursor proteins p105 and p100, their products p50 and p52, as well as phospho-Ser-866/870-p100 and phospho-Ser-933-p105 were analyzed in whole extracts by western blot (WB). CDK4 was used as loading control. Right: EMSA analysis of L1236 whole cell extracts after KDs of single NF-κB subunits and double KD of p50 and p52. Control of KD efficiencies is shown in Additional file 2: Figure S1B. **b** WB analysis of nuclear (N) and cytoplasmic (C) distribution of NF-κB subunits in HL cell lines, as indicated (top labels). p105 and PARP1 serve as purity controls for nuclear and cytoplasmic extracts, respectively. **c** Immunohistochemical detection of p105/p50 and p100/p52 in representative biopsies from HL patients. Arrows indicate nuclear abundance of p50 (left panel) and p52 (right panel) in malignant Reed-Sternberg cells compared to surrounding benign cells. A total of 20 biopsies were used, all were stained for p105/p50 and 18 biopsies were stained for p100/p52

To test the relative contribution of canonical and non-canonical NF-κB dimers in HL cells, we depleted NIK, a central component of non-canonical NF-κB signaling. Notably, NIK depletion not only affected processing of p100 but also of p105 (Fig. 1a). Ablation of NIK caused a severe reduction of C-terminal p100 and p105 phosphorylation, accumulation of the precursors, and decreased generation of their products p52 and p50 (Fig. 1a). This is similar to the NIK-dependent coupled generation of p50 and p52 that we recently demonstrated for non-canonical LTβ signaling in fibroblasts [4]. The p50 and p52 subunits are pivotal to the total constitutive NF-κB activity in HL cells, since RNA-mediated knockdown (KD) of both *NFKB1* (encoding p105/p50) and *NFKB2* (p100/p52) strongly affected global NF-κB DNA-binding, while depletion of the single NF-κB subunits, including RelA and RelB, did not (Fig. 1a). Consistent with its role in p50 and p52 production, NIK depletion had a similar effect on total NF-κB DNA-binding (Additional file 2: Figure S1A). Of note, in addition to slower migrating heteromeric species in the DNA binding assays, abundant activity migrated at the position of p50 and p52 dimers (Fig. 1a).

We next investigated the cytoplasmic-nuclear distribution of the NF-κB subunits in various HL cell lines (Fig. 1b). A significant fraction of p50 and p52 but only a low amount of RelA was present in the nucleus. Nuclear RelB was ubiquitously detected with variable intensities, whereas differing concentrations of nuclear c-Rel were observed throughout the cell lines. To evaluate the canonical and non-canonical NF-κB dimer composition, p50 and p52 were immunoprecipitated from nuclear extracts (Additional file 2: Figure S1C). We could readily detect p52-RelB and to a lesser extent p50-RelB dimers in all cell lines. However, p50-RelA dimers were present at variable lower amounts, and p50-c-Rel dimers were only abundant in L428 and KM-H2 cells, correlating with their generally restricted nuclear abundance. Furthermore, p50-p52 heterodimers were detected.

Nuclear enrichment of p50 and p52 was also evident in primary HL samples. A prominent nuclear staining was seen in 60 % of patients for p50 and in 83 % for p52 (Fig. 1c and Additional file 3: Table S1). Since the N-terminal epitope-directed antibodies recognize cytoplasmic precursors and the products, an excess of nuclear p50 and p52 is indicated in these cells. Altogether, these results demonstrate that non-canonical, NIK-driven IKK signaling predominantly accounts for constitutive NF-κB activity in HL cells, which is strongly dominated by production of dimers that contain p50 or p52. Compared to this, canonical p50-RelA heterodimers are under-represented.

### Constitutive activation of canonical and non-canonical NF-κB species generates largely overlapping cistromes

To investigate selective functional roles of the RelA, RelB, p50, and p52 subunits and of the prototypic p50-RelA and p52-RelB heterodimers in the gene expression repertoire of HL cells, we first performed ChIP-seq analyses of the four subunits to determine the genome wide distribution of their binding sites. This should also reveal if distinct regions and sequences are preferentially bound by canonical and non-canonical NF-κB species. The number of enriched regions greatly varied, up to 20-fold comparing RelA and p50 (Additional file 2: Figure S2A and Additional file 4: Table S2), reflecting the relative nuclear amounts of the respective NF-κB subunit (Fig. 1b). For each of the NF-κB datasets individual regions were selected and validated by qPCR (Additional file 2: Figure S2B). In each dataset the maximum frequency of ChIP-seq regions was observed around the TSS (Additional file 2: Figure S2C). The genomic locations of ChIP-seq regions in relation to annotated genes are representatively shown for p52 and p50. About half of the regions were located within transcribed gene regions, with a high percentage within introns (Additional file 2: Figure S2E).

**Fig. 2:**
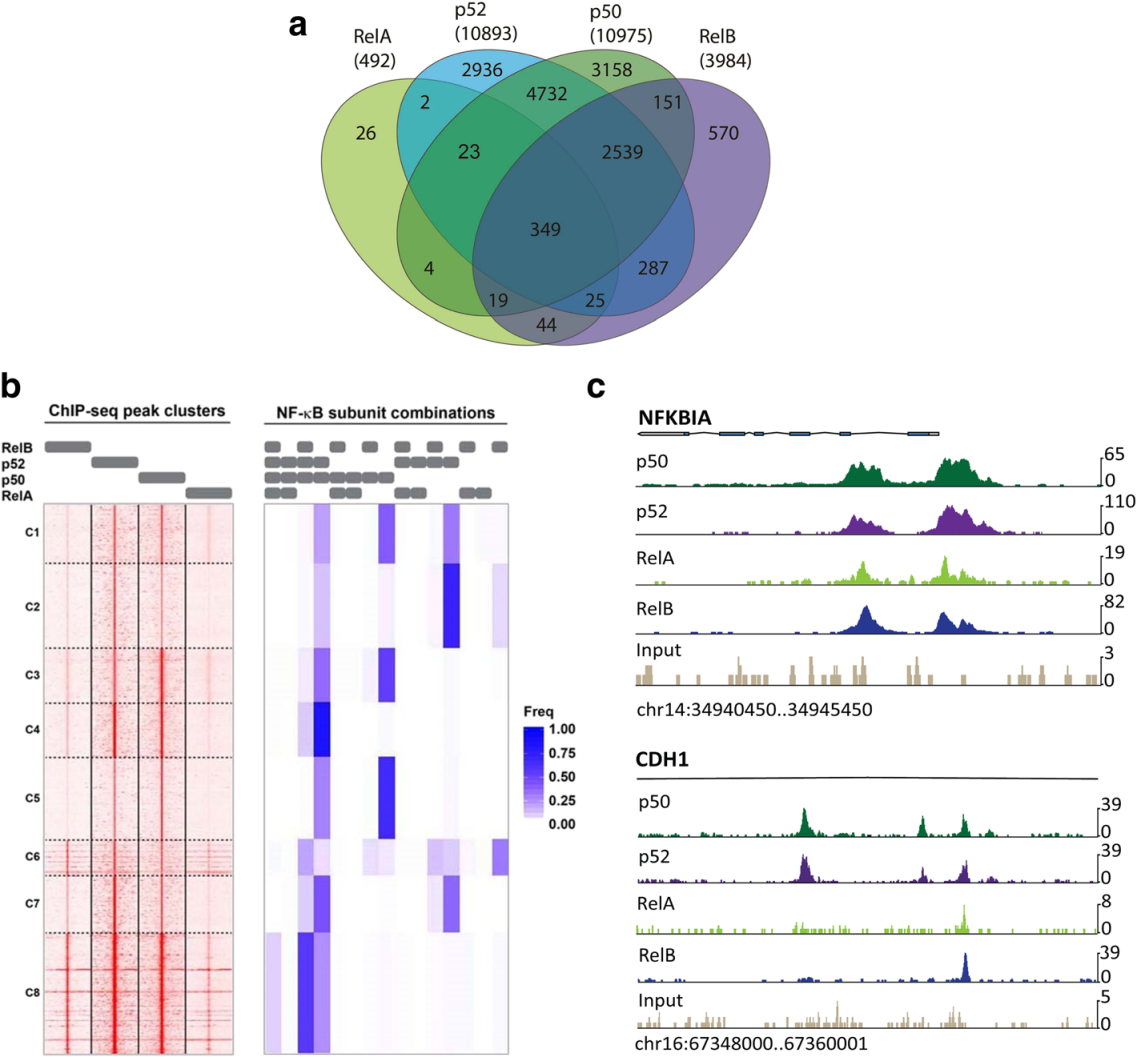
ChIP-sequencing analysis of genome wide distribution of RelA, RelB, p50, and p52 binding regions. **a** Venn diagram showing the total number and intersections of ChIP-seq regions for p50, p52, RelA, and RelB in L1236 cells. See Additional file 4: Table S2 and Additional file 2: Figure S2A for ChIP-seq data and gene assignments. **b** Genome-wide NF-κB subunit occupancy profiles. Left: Regions (peak summit + -500 bp) were grouped based on their profiles of ChIP enrichment over input for all four subunits using k-means clustering (k = 8). Gray bars on top indicate the ChIPed subunit. Each row represents a region that was identified as a peak for at least one of the four subunits. The distinct clusters are indicated by numbers on the left. Right: Regions were also classified by the combination of subunits based on the peak calling results. Gray bars on top indicate which combination of subunits was called. The color-coded heatmap shows the percentage of regions in each cluster with a specific combination of subunits. **c** Visualization of representative ChIP-seq regions (UCSC genome browser). Top, *NFKBIA* gene with overlapping peaks for p50, p52, RelA, and RelB in the promoter and first intron. Bottom, intron 2 of the *CDH1* gene showing selective binding of p50 and p52 to two ChIP-seq regions

Comparison of the cistromes obtained for the single NF-κB subunits revealed multiple intersections (Fig. 2a). RelA shares more than 94 % and RelB more than 85 % of their ChIP-seq regions with at least one other NF-κB subunit. Binding regions for p50 and p52 overlapped by about 70 %. Remarkably, a large fraction (41 %) of binding regions recruits p50 or p52 alone. Occupancy profiling reveals that combinatorial NF-κB subunit recruitment can be grouped into eight clusters (Fig. 2b) with a most prominent contribution of p50 and p52, either alone or in combination. Interestingly, RelB is most frequently seen in combination with p50 and p52, rather than with p52 alone, while RelA occupancy is least frequent. In many cases the tag pattern for all NF-κB subunits is very similar and binding regions are shared, even though the enrichment for RelA is weaker than that for p50, p52 and RelB (for example, *NFKBIA* gene). In other cases, the tag patterns differ and only subsets of subunits are present in selected ChIP-seq regions (*CDH1* gene, Fig. 2c).

**Fig. 3:**
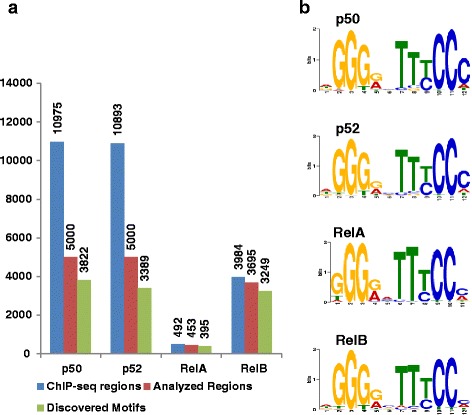
MEME *de novo* motif analysis revealed redundant consensus sites for each NF-κB subunit. **a** Occurrence of *de novo* detected NF-κB-like motifs in the indicated numbers of ChIP-seq regions analyzed by MEME. **b** NF-κB motifs detected by MEME *de novo* search in each of the four NF-κB subunit datasets of L1236 cells. See Additional file 5: Table S3 for CLOVER motif analysis data

Redundancy in DNA binding was also indicated by *de novo* motif searches using MEME [22] with the ChIP-seq datasets for the different NF-κB subunits. In all datasets, an NF-κB motif that resembled the known JASPAR database entries (MA0101; MA0107; MA0105) was found with very high frequency (Fig. 3a and b). To assess whether p50 and p52 dimers recognize distinct DNA motifs, MEME *de novo* search was performed with ChIP-seq regions that selectively recruited p50 or p52. Again, the NF-κB motifs found for each factor (Additional file 2: Figure S3) did not grossly differ from motifs found within regions that recruit more than one subunit (Fig. 3b). However, p50-selective motifs contained a higher conservation of a G in the first position compared to p52-selective motifs, indicating a putative contribution of highly symmetric binding sites for p50 homodimer recruitment.

We next searched for co-occurring binding sites of potential cooperating TFs within the subunit-specific ChIP-seq regions. Apart from the expected, statistically significant occurrence of the NF-κB motif in all datasets, we found an enrichment of AP-1, IRF, ETS, FOX, and CTCF motifs (Additional file 5: Table S3). This is in agreement with our finding that protein-occupied AP-1 and IRF motifs occur closely spaced to NF-κB motifs in global DNAse I hypersensitive site (DHS) mapping in HL cells [19]. Furthermore, we showed before that constitutively activated AP-1 and IRF5 in HL cells functionally synergize with NF-κB [19, 23].

The cistromes of NF-κB subunits in HL cells differ significantly from the binding landscapes previously reported for EBV-transformed lymphoblastoid B cells (LCL), where the membrane protein LMP1 constitutively activates the canonical and non-canonical NF-κB pathways [12]. In particular, the relative recruitment of RelA and RelB is notably less in HL cells compared to LCLs, while p50 and p52 are prevalent in HL cells. The ChIP regions of NF-κB subunits in HL cells overlap with those in LCL in average by 54 % (Additional file 6: Table S4). The binding regions that overlap in both cell types are summarized in Additional file 7: Table S5.

We also compared the combinatorial binding pattern of NF-κB subunits in ChIP-seq regions between HL and LCL (Additional file 8: Table S6). Both cell types strongly differed in combinatorial subunit binding, roughly in accordance with the differences in frequencies of subunit recruitment noted above. Interestingly, patterns exclusively containing the prototypic heterodimers p50-RelA or p52-RelB are rare in both cell types, about 2 %. The most frequent subunit combinations in HL cells contain p50 and/or p52, in contrast to RelA and RelB in LCL. Furthermore, as expected, the cistromes of the NF-κB species in HL cells and the DHS sites that we mapped in these cells [19] largely overlapped. Similarly, binding regions and DHS sites reveal an equivalent extent of overlap in LCL (Additional file 6: Table S4).

Thus, globally, the different NF-κB subunit recruitment in HL and LCL cells is in part dictated by the only partially overlapping open chromatin landscape of the two cell types.

### Distinct biological processes are differentially associated with genes that are up- or downregulated by p50/RelA and p52/RelB

**Fig. 4:**
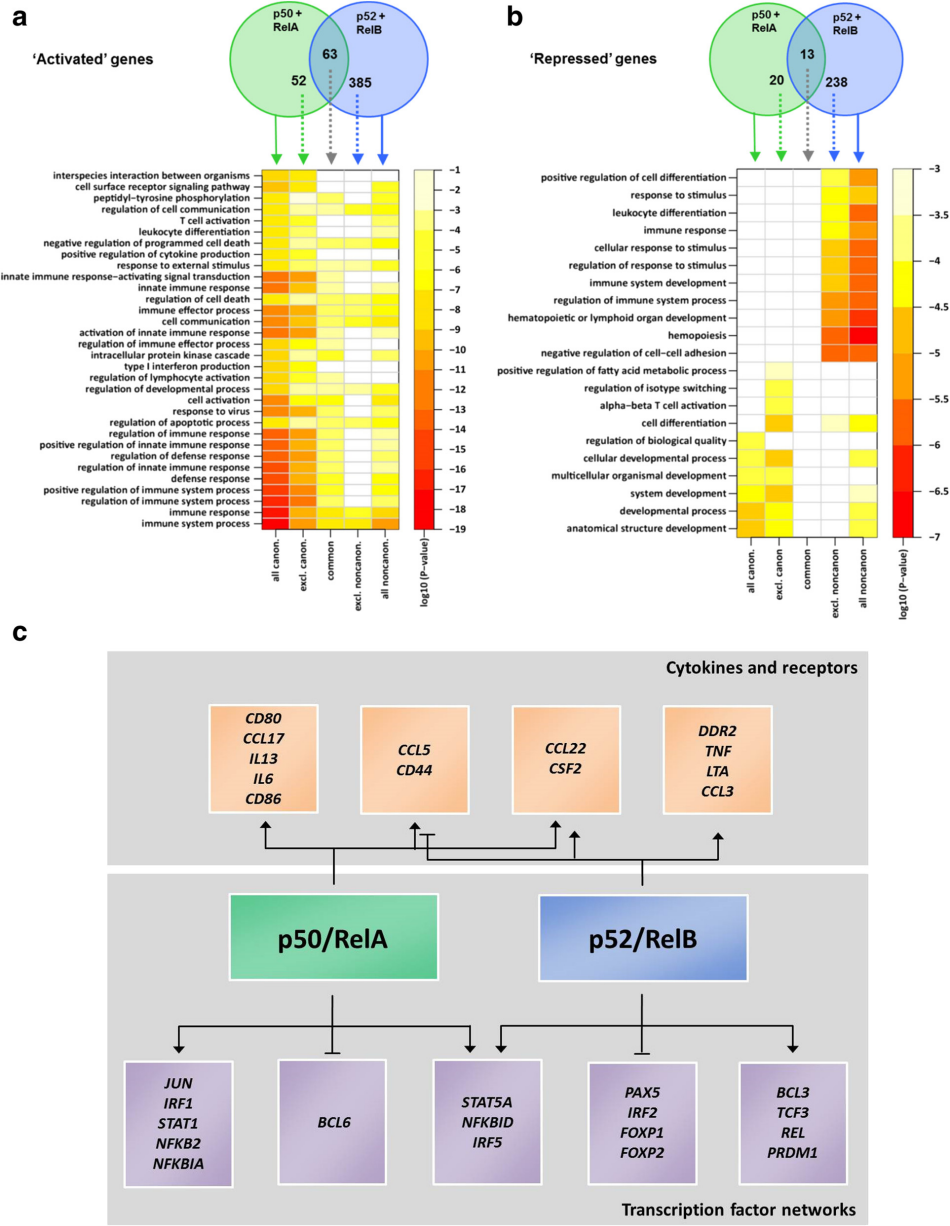
Enriched biological processes and genes with significance for HL biology controlled by p50/RelA and p52/RelB. **a**, **b** Genes activated (**a**) or repressed (**b**) by p50/RelA or p52/RelB in L1236 cells, respectively, and functional enrichment analysis of using Gene Ontology for biological processes (GO) analysis. Top panels: Venn diagrams displaying numbers of total, unique, and overlapping target genes for the two dimer combinations used for GO analysis (solid and stippled arrows, respectively). Direct target genes of p50/RelA and p52/RelB were defined as bound by at least p50 and p52, respectively, by ChIP-sequencing and differentially regulated upon KD of the specific dimers (FDR <0.05), and at least 10 % expression difference between the knockdown and control experiment). See Additional file 10: Table S8 for GO analysis data. **c** Schematic representation of canonical and non-canonical target genes that are known to be relevant for HL biology. Among both up- and downregulated genes, two functional groups were highly represented in HL literature: (1) cytokines and receptors, and (2) components of NF-κB-subordinated pathways [16, 19, 29, 50]

To determine the transcriptomes regulated by canonical and/or non-canonical NF-κB dimers, we depleted the subunit combinations p50/RelA and p52/RelB (Additional file 2: Figure S4A). Concentrations of siRNAs and treatment time schedules were optimized for each NF-κB subunit for efficient depletion (Additional file 2: Figure S4B). Samples were collected 1 day after the end of the respective siRNA treatments and one day before the initial onset of apoptosis. We performed global microarray expression analyses and determined direct targets by matching gene regulation with ChIP-seq binding data (Additional file 9: Table S7). Almost fivefold more genes were regulated by p52/RelB than by p50/RelA. While the majority of genes were transcriptionally activated, surprisingly, 22 % of p50/RelA and 36 % of p52/RelB regulated genes were down-regulated (Fig. 4a and b, top panels, Additional file 9: Table S7).

Alterations of gene regulation could have been caused indirectly by depletion of p100 and p105, which can sequester other NF-κB subunits in the cytoplasm and consequent liberation of bound Rel factors. However, no change in the nuclear patterns of RelA, RelB, or c-Rel was observed (Additional file 2: Figure S4C), suggesting that the altered expression of genes is a direct consequence of the KD of NF-κB subunits and not of a loss of the IκB function of the precursors.

For up- and for downregulated genes, the p50/RelA- and p52/RelB-dependent sets in each case partially overlapped, but also revealed genes that were only controlled by either p50/RelA or p52/RelB (Fig. 4a and b, top panels). These datasets contained a considerable number of novel direct NF-κB targets, but also previously known NF-κB target genes, as expected (Additional file 9: Table S7). A number of genes that are differentially up- or downregulated by p50/RelA- and/or p52/RelB have a high biological significance for HL (for a selection, see Fig. 4c) and encode cytokines and chemokines responsible for clinical symptoms and TF networks implicated in this disease [16].

We next categorized genes directly regulated by p50/RelA and/or p52/RelB according to their activation or repression status and grouped them by related biological processes, using Gene Ontology (GO) terms. The set of genes upregulated by p50/RelA was predominantly enriched in immune system processes. These genes are also important for cell activation, cell communication, and regulation of programmed cell death. The set of genes upregulated by p52/RelB is also enriched in these categories, however, covering a broader spectrum of biological functions. The cell death categories are shared by both subunit combinations (Fig. 4a) and include known NF-κB induced anti-apoptotic genes such as *CFLAR* and *BCL2L1* (Additional file 9: Table S7).

The gene sets that were downregulated by p50/RelA or p52/RelB (Additional file 9: Table S7) differed greatly in the enriched GO terms (Fig. 4b, Additional file 10: Table S8). Genes that were selectively downregulated by p50/RelA, like *BCL6*, are mainly related to general cellular developmental and differentiation processes. The genes exclusively downregulated by p52/RelB on the other hand, including *FOXP1* and *LEF1*, are highly enriched in terms such as hematopoietic and lymphoid organ development, negative regulation of cell adhesion, and positive regulation of cell differentiation (Fig. 4b and c, Additional file 9: Table S7).

Collectively, these results show that p52/RelB controls the majority of the NF-κB regulated genes and especially represses genes involved in differentiation processes. However, both p50/RelA and p52/RelB control genes that are involved in modulation of immune system responses as well as cell survival. Both pathways are differentially engaged in the regulation of genes with previously documented high or low expression in HL (Fig. 4c).

### Correlation of combinatorial binding patterns of NF-κB subunits and gene expression regulation

**Fig. 5:**
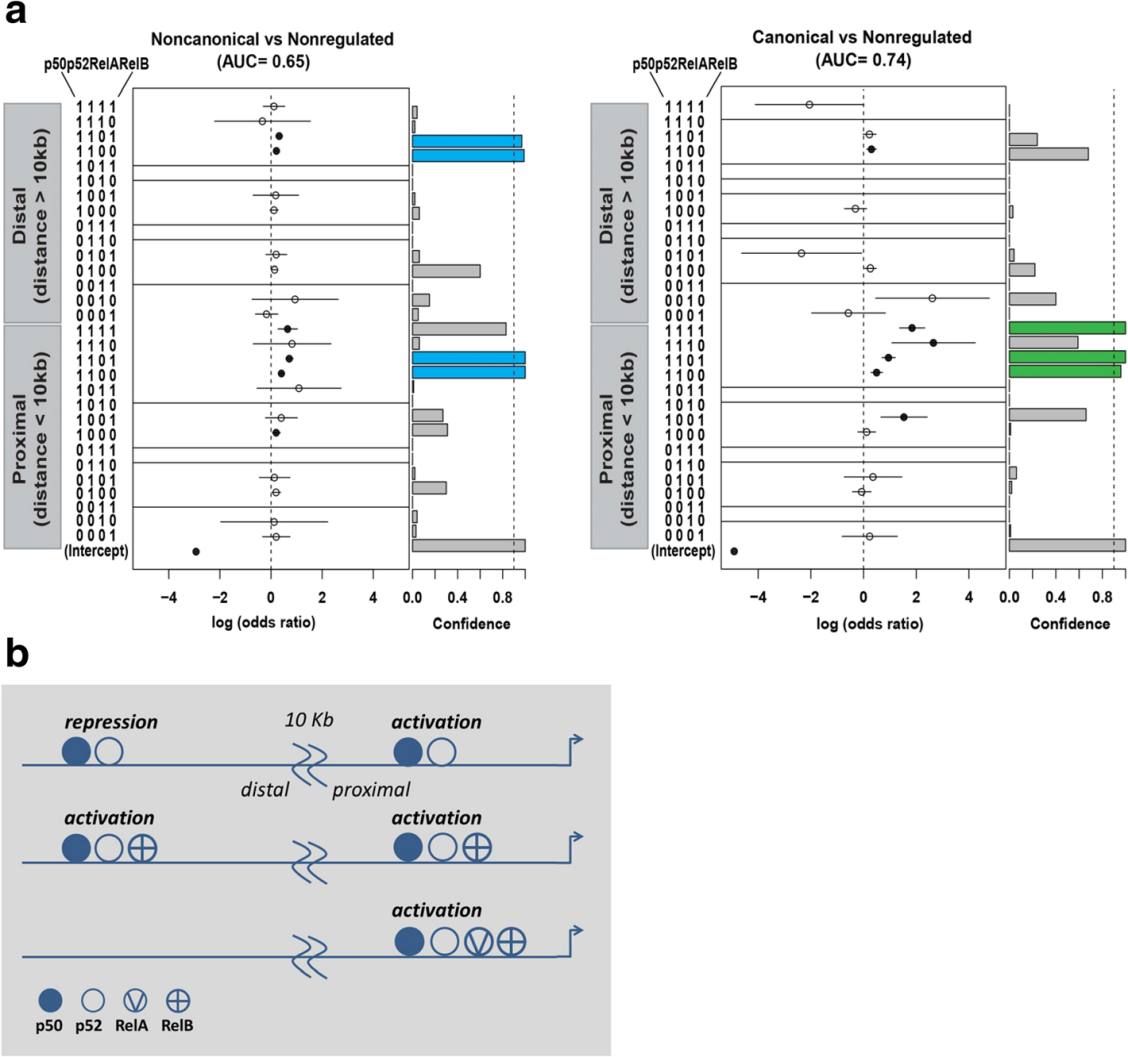
Prediction of gene expression patterns from combinatorial binding of NF-κB subunits using logistic regression analyses. **a** Left: Regulation by non-canonical NF-κB versus no regulation. Right: Regulation by canonical NF-κB versus no regulation. Note that both, up- and downregulated genes are included in the categories ‘canonical’ regulated or ‘non-canonical’ regulated genes. Different parameter choices were systematically analyzed to evaluate the performance of the prediction framework for the logistic regression models (see Additional file 2: Figure S5). The y-axis shows the combinations of NF-κB subunits, which are listed as binary vectors (1 = presence, 0 = absence). The x-axis shows parameter estimates of the logistic regression models, which can be interpreted as log odds ratios and are indicated by circles. Vertical lines show confidence intervals (95 %). Filled circles indicate that parameters are significantly different from zero (*P* <0.01). Missing estimates and confidence intervals occur when the specific subunit combination was not observed in the dataset. Bar plots on the right side indicate the percentage of bootstrap samples in which each parameter was significantly different from zero (*P* <0.01). The dashed line marks 90 %. See Additional file 1: Supplemental Experimental Procedures for details. **b** Schematic presentation of the dependency of gene activation or repression on the distance and subunit occupancies of NF-κB binding regions

By fitting logistic regression models (see Additional file 1: Supplemental Experimental Procedures for details) we analyzed if specific combinations of NF-κB subunits that were determined within the individual ChIP-seq regions could be used to predict modes of gene expression regulation by canonical and non-canonical dimers. The best model parameters were estimated, also considering the available L1236-DHS data and the genomic 3D architecture in LCL [19, 24], as outlined in Additional file 2: Figure S5A. We found that binding of both, p50 and p52, either alone or in combination with RelB or RelB plus RelA was significantly associated with regulation (that is, activation or repression) of gene expression by canonical and non-canonical dimers. Specifically, the genes regulated by p52/RelB revealed a high preference for binding of p50 along with p52, with or without RelB and were independent of proximal or distal localization of the region relative to the TSS (Fig. 5a). Genes sensitive to p50/RelA knockdown also revealed the same preference for p50/p52/RelB but the most significant subunit combination was p50/p52/RelA/RelB (Fig. 5a). In this case all regions were restricted to proximal positions. Likewise, proximal binding of all four subunits, thus including RelA, was highly predictive for genes regulated by canonical compared to non-canonical dimers (Additional file 2: Figure S5B). Furthermore, we identified the preferential NF-κB subunit combinations associated with gene activation or repression. p50/p52/RelB and p50/p52/RelA/RelB were significantly associated with activated genes (Additional file 2: Figure S5C). Interestingly, although with lesser confidence, binding of p50/p52 scored highest with repressed genes when in distal positions, but with activation, when at proximal positions (Additional file 2: Figure S5C and D). The compositional and positional correlation of NF-κB subunit binding pattern with gene activation and repression is schematically summarized in Fig. 5b. With exception of p50/p52 (binary code ‘1100’ in Fig. 5a), the identified high confidence binding patterns did not simply correlate with their frequency of occurrence in the ChIP-seq datasets (Additional file 8: Table S6). Importantly, exclusive recruitment of prototypic dimers (p50-RelA or p52-RelB) was not significantly associated with gene regulation (‘1010’ and ‘0101’ in Fig. 5a and Additional file 2: Figures S5B and C).

Taken together, the logistic regression analyses reveal that the specificity of NF-κB subunit-selective gene regulation is not determined by the exclusive recruitment of the targeted prototypic dimers, but by more complex combinations of NF-κB subunits, involving a parallel contribution of p50 and p52. The most active combinations (1111, 1101, and 1100) are part of clusters C4 and C8 (Fig. 2b), revealing strongest and equal p50 and p52 recruitment.

### Cell survival of HL cells is predominantly controlled by the non-canonical NF-κB pathway

**Fig. 6:**
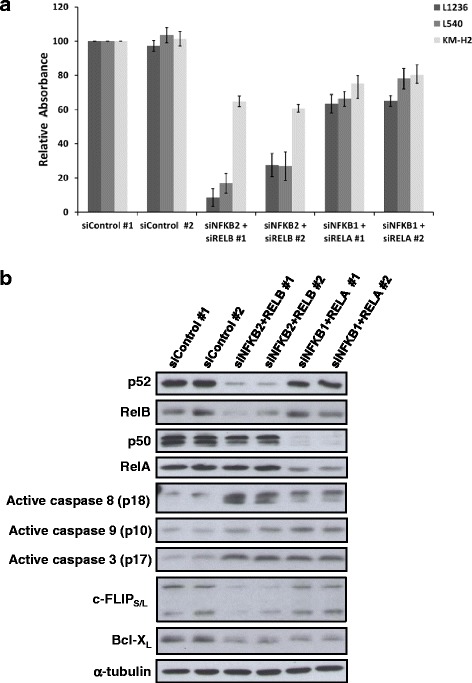
Non-canonical as well as canonical subunits inhibit apoptosis of HL cells. **a** HL cell lines (L1236, L540 and KM-H2) were treated with two distinct siRNA sequences against p50/RelA and p52/RelB, as indicated and harvested 3 days after the end of the siRNA treatments. Overall redox activity of the cells was measured using Alamar Blue assay. Error bars represent SEM (n = 3). See Additional file 2: Figures S6A and B for time-course experiments. For time-course experiments see Additional file 2: Figures S6A and B. **b** WB analysis of p50/RelA or p52/RelB KD efficiencies and expression of the initiator caspase 8 (p18), the initiator caspase 9 (p10), and the effector caspase 3 (p17) in the samples of L1236 cells treated with siRNAs as described above. Protein levels of the two apoptosis inhibitors (c-FLIP_S/L_ and Bcl-X_L_) identified as NF-κB target genes are also shown. α-tubulin was used as loading control

We next investigated the relative impact of p50-RelA and p52-RelB, both of which activated genes associated with cell death/apoptosis (Fig. 4a), on cell viability. Close monitoring of siRNA-treated HL cells revealed a 30 % to 40 % decrease of cell viability upon p50/RelA KD and up to 95 % reduction in viability upon p52/RelB KD (Fig. 6a and Additional file 2: Figures S6A and B). The cleavage of initiator caspases 8 and 9, indicating apoptosis, were predominantly increased upon KD of non-canonical and canonical subunits, respectively, while effector caspase 3 was equally activated in both groups (Fig. 6b). Interestingly, we observed a correlation between the activation of initiator caspases and the protein amounts of negative regulators of apoptosis such as c-FLIP and Bcl- X_L_ (Fig. 6b). c-FLIP downregulation sensitizes HL cells to death receptor-induced apoptosis [25]. *CFLAR* mRNA expression was downregulated by the KD of both combinations of NF-κB subunits, but a decrease in protein levels was only observed upon the KD of p52/RelB, which was concomitant to strong caspase 8 activation (see Fig. 6b). Overexpression of Bcl-X_L_ blocks the intrinsic apoptosis pathway in many cell types [26, 27] and its protein level was downregulated by the KD of p52/RelB as well as of p50/RelA (Fig. 6b). To investigate the possible connection between the downregulation of Bcl-X_L_ caused by the KD of NF-κB dimers and apoptosis, we tested the effect of *BCL2L1* knockdown. Following this, we observed an up to 30 % decrease in cell viability in HL cell lines, along with activation of caspases 9 and 3 (Additional file 2: Figure S6C and D). This suggests that Bcl-X_L_ blocks the activation of the intrinsic apoptosis pathway in HL cells and is partially responsible for the NF-κB-dependent control of HL cell survival.

Taken together, our results indicate that the activation of p52-RelB is essential for the survival of HL cells through the upregulation of proteins that block the intrinsic and especially the extrinsic apoptosis pathway (for example, c-Flip), whereas p50-RelA also contributes to survival by the upregulation of Bcl-X_L_ and subsequent inhibition of the intrinsic apoptosis pathway.

### p50-RelA and p52-RelB gene signatures distinguish classical Hodgkin lymphoma from other lymphomas and normal B cells

**Fig. 7:**
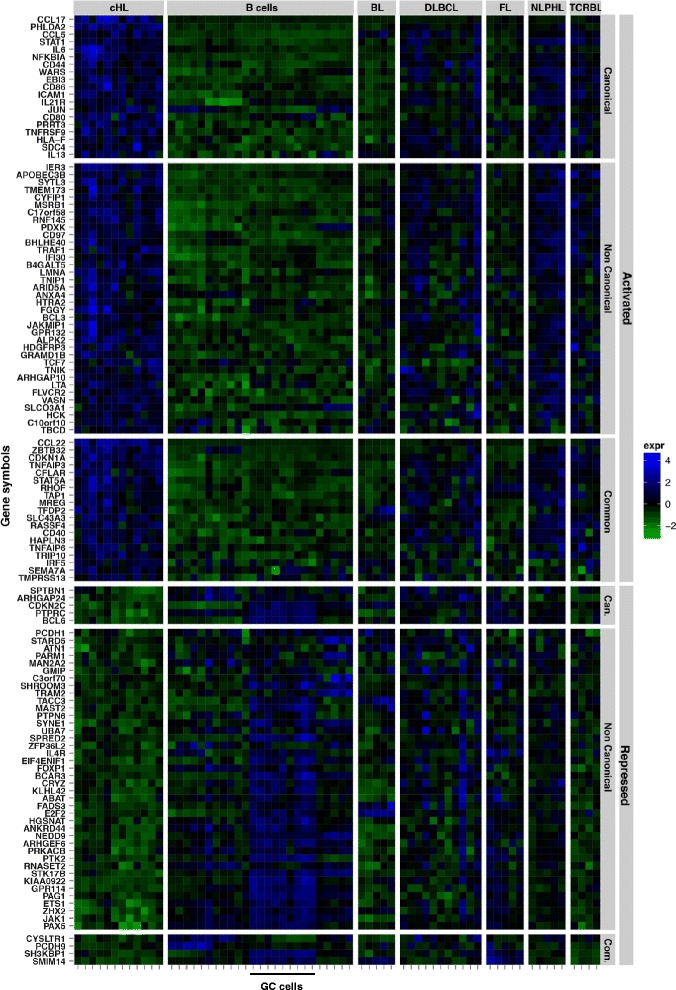
Canonical and non-canonical NF-κB target genes differentiate primary HL from other lymphoid malignancies. The canonical and non-canonical NF-κB signatures identified in L1236 cells were evaluated using public gene expression data from patient samples (normal and malignant B cells [28]) available in GEO (GSE12453), by comparing their expression in HL samples to the rest. The heat map expression levels are encoded by the base 2 logarithmic scale color bar (right). A total of 123 NF-κB regulated genes were differentially expressed in HL samples and presented concordant expression changes (activation by NF-κB dimers and upregulation in HL samples or repression by NF-κB dimers and downregulation in HL samples). The gene names (left) and lymphoid cell types (top) are indicated. BL, Burkitt’s lymphoma; DLBCL, diffuse large B-cell lymphoma; FL, follicular lymphoma; NLPHL, nodular lymphocyte-predominant HL; TCRBL, T cell-rich B cell lymphoma. Germinal center (GC) B cells are indicated (bottom). See also Additional file 11: Table S9

In order to compare the gene sets regulated by the p50/RelA and p52/RelB subunit combinations, reflecting canonical and non-canonical NF-κB signaling in HL cells *in vitro*, with primary human lymphoma, we performed a retrospective analysis of microarray datasets generated from micro-dissected Hodgkin/Reed Sternberg (HRS) cells of classical HL, other malignant B cell lymphomas, and normal B cells at various stages of differentiation [28, 29]. We identified 123 genes that were direct NF-κB targets in L1236 cells and differentially regulated in primary HRS cells when compared to other malignant and normal B cells (Fig. 7; Additional file 11: Table S9). These genes were classified into three groups, genes regulated by canonical, non-canonical, or both NF-κB pathways. Selected examples of regulated genes were validated by RT-qPCR (Additional file 2: Figure S7).

Some genes that were activated by NF-κB in L1236 cells and identified as classical HL signatures were also upregulated in other B-cell lymphomas, where NF-κB activation has been observed previously, such as nodular lymphocyte-predominant HL (NLPHL) [28], T cell-rich B cell lymphoma (TCRBL) [28] and DLBCL [30] (Fig. 7). These genes were downregulated in normal B cells and also in Burkitt’s lymphoma (BL), where NF-κB is not activated [31, 32].

Interestingly, genes that were repressed in L1236 by NF-κB and downregulated in classical HL, were upregulated in normal germinal center (GC) B cells (centroblasts and centrocytes) (Fig. 7). Thus, NF-κB might contribute to the characteristic de-differentiation of HL cells, which are derived from germinal center B cells [16].

Our results show that gene sets that are differentially up- or downregulated by canonical and non-canonical NF-κB signaling in L1236 cells, which were used as a model system, are differentially expressed in a similar manner in primary HL tumor cells compared to other B cell malignancies.

## Discussion

In various lymphoid malignancies, oncogenic constitutive NF-κB activity is determined to varying extents by canonical and non-canonical signaling, as indicated by mutations in upstream pathway components [13]. However, the specific contributions of constitutive canonical versus non-canonical NF-κB species to cancer biology remain poorly understood. Here, we analyzed the genomic landscapes of p50, p52, RelA, and RelB in HL cells by ChIP-sequencing and combined these data with the genes regulated by these subunits.

We could demonstrate that NF-κB dependent gene expression in HL cells is largely driven by the non-canonical pathway. The central kinase of this pathway, NIK, is known to be overexpressed in HL cell lines and primary cells [18, 33]. We show that NIK triggers not only p100, but also p105 precursor processing to p52 and p50. This finding is consistent with NIK-dependent processing of both precursors in LTβ-stimulated cells, which we have recently characterized [4]. We also noted a surprisingly low translocation of RelA. Interestingly, low levels of nuclear translocation/activation of RelA and the parallel increase of p50 and p52 in HL cells can be observed at late time points upon stimulation with TWEAK or CD40 [34, 35], suggesting that this might be a common feature of cells with stimulated NIK pathways. A similar pattern is also seen in peripheral T cell lymphoma [36] and multiple myeloma [37].

Constitutive non-canonical signaling causes abundant nuclear activities of p50 and p52 and each of them is recruited to more than 10,000 binding regions. This number of binding regions for NF-κB subunits in HL cells is comparable to the number determined in prior studies using lymphoblastoid B cells [10, 12]. However, we show that only a small fraction of these genes are transcriptionally regulated. In addition, we observed a lower contribution of RelA recruitment and of p50-RelA to the control of NF-κB dependent gene expression.

We asked for non-redundant functions, that is, a functional ‘addictiveness’ of regulated genes to particular subunit combinations by knocking down p50/RelA or p52/RelB. Several established NF-κB targets, which were previously identified [38], such as *CCR7*, *CCND2*, *FAS*, *JUNB*, or *VCAM1*, were bound by canonical and non-canonical subunits, but not regulated upon knockdown. This fact can be explained by redundant functions of NF-κB subunits or the applied stringency of the parameters used in our gene expression analysis. From a total of 501 activated genes, 63 were activated by both, p50/RelA and p52/RelB, revealing additive or synergistic regulation. This group of target genes comprised *BIRC2* (encoding cIAP1), *BCL2L1* (Bcl-X_L_), *CFLAR* (c-FLIP), *CD40*, *IRF5*, *STAT5A*, *CSF2*, *CCL22*, and *HEY1*, all of which are known to be expressed at high levels in HL cells [19, 38, 39].

Surprisingly, one-third of all genes regulated by NF-κB were suppressed. The observed suppression of genes by both dimer combinations may be caused by subunit exchange mechanisms. p50 or p52 dimers that lack transactivation domains and do not interact with transcriptional co-activators, may prevent access of activating RelA or RelB containing heterodimers. Furthermore, p50 and p52 may recruit repressor complexes, including histone deacetylases [1, 40, 41], in a context-dependent manner to suppress transcription. Indeed, our logistic regression models indicate that a combined recruitment of p50 and p52 significantly correlated with gene expression.

In addition to the separate biological processes identified by GO analyses for canonical and non-canonical subunit combinations, overlapping functions were implicated in cell death regulation. Indeed, we were able to demonstrate that apoptosis was most prominently inhibited by p52/RelB that primarily affected the extrinsic apoptosis pathway, while p50/RelA contributed to cell death protection by interfering with the intrinsic pathway. Thus, HL cells engage in both apoptosis pathways to block cell death, which is similar to mature murine B cells, where both are essential for maintenance and in part are interchangeable [42].

Another interesting observation was the fact that genes downregulated by NF-κB in HL cells were upregulated in normal germinal center (GC) B cells (Fig. 7). Among these genes were *BCL6* and *PAX5* as well as other important regulators of B cell development, such as *FOXP1* [43], *JAK1* [44], and *PTPN6* [45], suggesting that NF-κB signaling may contribute to the known de-differentiation process of HL cells, which are derived from GC cells [16]. Activation of the canonical NF-κB subunits is important for the maintenance and differentiation of GC B cells [46], but the role of the non-canonical subunits in this stage of the B-cell development remains unclear. Our signatures of suppressed genes will thus be an interesting source for future studies of HL lymphomagenesis.

We also show that the differential gene regulation programs of canonical and non-canonical NF-κBs were neither explainable by differences in their DNA sequence elements, nor by exclusive recruitment of dimer species to the genes they regulate. The MEME analysis of the ChIP-seq datasets for the different NF-κB subunits revealed high redundancy in the conserved motifs. Not taking into account a very weak first position in motifs related to p50, p52, and RelB, we have found in this study very similar 11-bp motifs for each factor (p50, p52, RelA, and RelB). Yet, we could notice a more specific C at the final position for p50 and p52. A similar redundancy of motifs was also found in the recruitment patterns in lymphoblastoid cells [12].

The motifs found, for example, in regions selective for p50 or p52 did not considerably differ from those associated with additional binding of RelA or RelB, which indicates that other constraints such as protein-protein interactions are important in determining recruitment. Accordingly, the differential interaction of NF-κB with other TFs may establish functional gene selectivity. Motifs for bZIP, ETS, FOX, IRF and CTCF families were significantly over-represented in binding regions of all four NF-κB subunits. Interestingly, these motifs belong to a dense cluster of TF binding sites surrounding cohesin anchor sites, which may contribute to the regulation of nearby genes [47]. Indeed, we have shown before that AP1 and IRF5, which are recruited to bZIP and IRF motifs, functionally synergize with NF-κB [19, 23]. This synergism may control chromatin accessibility, as was shown for AP1 in other systems [48, 49].

It remains a difficult task to correlate binding of a given subunit to an individual gene with the regulation of this gene, since regulation may be conferred by other remote regions. We therefore developed logistic regression models to dissect the overall correlation between transcriptional regulation and recruitment of combinations of NF-κB subunits. This analysis provided insight into the functional subunit organization, such as a cross-contribution of p50 to gene regulation by non-canonical p52-containing dimers and vice versa. It ruled out that exclusive recruitment of the prototypic dimers p50-RelA and p52-RelB alone is significantly associated with gene regulation. This analysis also clearly indicated that RelA-containing binding patterns correlated with function only from proximal, but not distal localizations, while p50, p52, and RelB were distance independent. Consequently, the distinct NF-κB species do have different preferences for action through enhancers or other remote positions versus promoters. Thus, the models developed here using binary codes for NF-κB subunits allowed us to correlate gene expression regulation with specific combinatorial subunit occupancy.

## Conclusions

In summary, in this study we have dissected the NF-κB subunit dependent gene expression pattern in HL cells, providing datasets that will be the basis for future comparative analyses once equivalent functional investigations for other NF-κB-associated malignancies become available. The understanding of the differential contributions of canonical and non-canonical NF-κB species to transcriptional networks and of their potential synergisms of action on targets will be crucial for establishing refined functional gene signatures. The latter may provide important information for future stratification of NF-κB associated malignancies and for the evaluation of novel NF-κB pathway directed drugs for individualized therapies.

### Ethics approval and consent to participate

The use of human material was approved by the Ethik-Kommission of the Charité, Universitätsmedizin Berlin (EA4/104/11) and was performed in accordance with the Declaration of Helsinki. Informed consent was obtained from all subjects, where required.

### Availability of data and materials

Geo accession numbers for ChIP-seq and gene expression data are GSE63736 and GSE64232, respectively.

## Additional files

Additional file 1: Supplemental Experimental Procedures shows a detailed version of materials and methods. (DOCX 60 kb) Additional file 2: Figure S1 shows co-immunoprecipitation of p50 and p52 with NF-κB subunits. **Figure S2** shows the number of ChIP-seq regions bound by the individual NF-κB subunits, the number of genes assigned to ChIP-seq regions, the validation of ChIP-seq regions by qPCR, the frequency of ChIP-seq regions for NF-κB subunits relative to the transcription start site (TSS), p52 recruitment to selected ChIP-seq regions upon treatment of L1236 cells with NFKB2-siRNAs and control-siRNAs, and the genomic locations of p50 and p52-ChIP-seq regions with respect to annotated genes. **Figure S3** shows p50- and p52-selective motifs detected by using MEME. **Figure S4** shows the optimization and specificity controls for gene knockdown experiments. **Figure S5** shows the prediction of gene expression patterns from combinatorial binding of NF-κB subunits. **Figure S6** shows cell death induction by depletion of p50/RelA, p52/RelB or Bcl-X_L_. **Figure S7** shows RT-qPCR analyses of genes that were differentially expressed in classical HL compared to other lymphoid malignancies and normal B cells. (PDF 3236 kb) Additional file 3: Table S1 shows immunohistochemical analyses of HL biopsy samples with p100/p52 and p105/p50 antibodies. (DOCX 12kb) Additional file 4: Table S2 shows ChIP-seq regions for RelA, RelB, p50, and p52 and assigned genes. (XLSX 5263 kb) Additional file 5: Table S3 shows motifs over-represented in NF-κB ChIP-seq regions found by CLOVER analysis. (XLSX 11 kb) Additional file 6: Table S4 shows the overlap of NF-κB subunit binding and DNAse I hypersensitive sites (DHS) in L1236 and GM12878 cells. (DOCX 13 kb) Additional file 7: Table S5 shows the overlap of DHS and ChIP-seq regions for RelA, RelB, p50, and p52 in L1236 and GM12878 cells. (XLSX 1227 kb) Additional file 8: Table S6 shows the comparison of combinatorial subunit binding between L1236 and GM12878 cells. (DOCX 13 kb) Additional file 9: Table S7 shows the gene level summary of gene expression results. (XLSX 3346 kb) Additional file 10: Table S8 shows the functional enrichment analysis of p50/RelA and p52/RelB targets using ‘Gene Ontology (GO) for biological processes’ analyses. (XLSX 6646 kb) Additional file 11: Table S9 shows p50/RelA and p52/RelB targets that are differentially expressed in primary samples of lymphoid malignancies and normal B lymphocytes. (XLSX 40 kb)

## Abbreviations

BL: Burkitt’s lymphoma
ChIP: chromatin immunoprecipitation
ChIP-seq: chromatin immunoprecipitation followed by sequencing
DHS: DNAse I hypersensitive site
DLBCL: diffuse large B-cell lymphoma
EBV: Epstein-Barr virus
GC: germinal center
GEO: gene expression omnibus
GO: gene ontology
HL: Hodgkin lymphoma
HRS: Hodgkin/Reed Sternberg
IKK: IκB kinase
IKKα: IκB kinase α
IP: Immunoprecipitation
IκB: inhibitor of κB
KD: RNA-mediated knockdown
LCL: lymphoblastoid B cells
LTβR: lymphotoxin-β receptor
MEME: multiple EM for motif elicitation
NF-κB: nuclear factor κB
NIK: NF-κB-inducing kinase
NLPHL: nodular lymphocyte-predominant HL
p50/RelA or p52/RelB: slash refers to combined or single function of subunits
p50-RelA or p52-RelB: hyphen refers to the indicated heterodimer
qPCR: quantitative real-time PCR
RT-qPCR: reverse transcription quantitative PCR
TCRBL: T cell-rich B cell lymphoma
TFs: transcription factors
TSS: transcription start site
TWEAK: TNF-related weak inducer of apoptosis
