# Supplementary material for: A roadmap of constitutive NF-κB activity in Hodgkin lymphoma: Dominant roles of p50 and p52 revealed by genome-wide analyses
Source: Genome Med. 2016 Mar 17;8:28. doi: 10.1186/s13073-016-0280-5 (PMC4794921; doi:10.1186/s13073-016-0280-5)
Supplement: Additional file 1: — Supplemental Experimental Procedures shows a detailed version of materials and methods. (DOCX 60 kb) [file 13073_2016_280_MOESM1_ESM.docx]

**Additional File 1: Supplemental Experimental Procedures**

**List of antibodies used in WB and immunohistochemistry**

| **Method** | **Protein target** | **Catalog number** | **Supplier** |
| --- | --- | --- | --- |
| **Western blot** | NIK | 4994 | Cell Signaling |
|  | pp105 (Ser933) | 4806 | Cell Signaling |
|  | p105/p50 | sc-8414 | Santa Cruz Biotechnology |
|  |  | 3035 | Cell Signaling |
|  | pp100 (Ser866/870) | 4810 | Cell Signaling |
|  | p100/p52 | 05-361 | Upstate |
|  | RelA | sc-372 | Santa Cruz Biotechnology |
|  | RelB | 4922 | Cell Signaling |
|  | c-Rel | sc-71 | Santa Cruz Biotechnology |
|  | Caspase 8 | sc-7890 | Santa Cruz Biotechnology |
|  | Caspase 9 | 9508 | Cell Signaling |
|  | Caspase 3 | 9662 | Cell Signaling |
|  | c-FLIP | sc-5276 | Santa Cruz Biotechnology |
|  | Bcl-xL | sc-8392 | Santa Cruz Biotechnology |
|  | α-Tubulin (loading control) | sc-5286 | Santa Cruz Biotechnology |
|  | CDK4 (loading control) | sc-260 | Santa Cruz Biotechnology |
| **Immunoprecipitation** | p105/p50 | sc-8414 | Santa Cruz Biotechnology |
|  | p100/p52 | 05-361 | Upstate |

**RNAi experiments.** Chemically synthesized siRNAs (designed and synthesized by Dharmacon, Lafayette, CO, USA) were passively transfected into HL cell lines using Accell delivery media (Dharmacon) and 1% FBS, according to the manufacturer’s instructions. The shortest period of siRNA incubation and the lowest siRNA concentration were chosen for each siRNA target. The following conditions were used in this study: *MAP3K14* (4 days, 1 µM) *NFKB1* (3 days, 1 µM), *NFKB2* (2 days, 500 nM), *RELA* (2 days, 500 nM), *RELB* (3 days, 1 µM), *BCL1L2* (3 days, 1 µM), and RIPK1 (4 days, 1 µM). Normal cultivation conditions were re-established after the transfection period. The number of days between the reestablishment of the FBS conditions and the harvesting of the cells are mentioned in the figure legends.

For the gene expression analyses, double knockdown experiments for *NFKB1*+*RELA* and *NFKB2*+*RELB* were performed independently in biological triplicates, using two distinct siRNA sequences for each target. For the simultaneously double KD, siNFKB2 and siRELA were added 1 day later to the samples that already contained siRELB or siNFKB1, respectively. Cells were harvested 1 day after the re-establishment of the normal FBS condition and one day before the initial onset of apoptosis. A list of the siRNA sequences is provided below.

**List of Accell siRNA sequences**

| **Gene symbol** | **Sequences (5’ → 3’)** |
| --- | --- |
| *NFKB1* | CCAAAGUAUAAAGAUAUUA |
|  | GGCUCAUGUUUACAGCUUU |
| *NFKB2* | UCAUUGAGCAGAUAGUCUA |
|  | CUGUCAAGAUCUGUAACUA |
| *RELA* | CUUCCAAGUUCCUAUAGAA |
|  | GGAUUGAGGAGAAACGUAA |
| *RELB* | GUAGGAUUCGGAAAAGAUU |
|  | UGGAGAUCAUCGACGAGUA |
| *BCL2L1* | GCCUUUGUUUUGAUGUUUG |
|  | CCAGGGAGCUUGAAAGUUU |
| *MAP3K14* | GUAUGGAGCUGUGAGAGAA |
|  | CAAGCUAUUUCAAUGGUGU |
| RIPK1 | CUUUCUUUACUAAGUGAUA |
| Non-targeting siRNA #1 | Catalog Item: D-001910-01-50 |
| Non-targeting siRNA #2 | Catalog Item: D-001910-02-50 |

**Protein extraction, cell fractionation, and western blotting.** To prepare whole protein extracts, cells were washed with 1X PBS and lysed with lysis buffer (20 mM HEPES pH 7.9, 350 mM NaCl, 0.5 mM EDTA, 0.1 mM EGTA, 1 mM MgCl_2_, 20 % glycerol, 1 % Nonident P-40 (NP-40), 500 µM Pefabloc, 3 mM NaF, 1 mM β-glycerophosphate, 1 mM DTT, 1 mM Na_3_VO_4_, and complete protease inhibitor cocktail (Roche)). After 20 min incubation at 4 °C, the lysate was centrifuged for 10 min at 14,000 RPM. Nuclear and cytoplasmic extract were prepared as previously described, [1] with minor modifications. Western blots (WBs) were analyzed by chemiluminescence, following the manufacturer’s recommendations (Millipore) or by home-made ECL solution as previously described [2].

**Immunoprecipitation.** One microgram of protein from nuclear extracts of HL cells was diluted in pull down buffer (20 mM Tris-HCl 7.4, 150 mM NaCl, 50 mM KCl, 1.5 mM MgCl_2_, 0.2 % NP-40, 0.2 mM EDTA, 25 % glycerol, 3 mM NaF, 1 mM β-glycerophosphate, 1 mM Na_3_VO_4_, 50 nM Calyculin A (Cell Signaling), 1mM DTT, 500 µM Pefablock (Roche), and protease inhibitor cocktail (Roche)) and pre-cleared with protein G for 30 min. Cleared lysates were incubated with either p50 or p52 antibodies and protein G sepharose beads (GE Healthcare). Immunocomplexes were precipitated overnight and analyzed by WB.

**Electrophoretic mobility shift assay.** Electrophoretic mobility shift assay (EMSA) was performed as described previously [3]. Briefly, H2K oligonucleotide probe was used and the DNA binding reactions were performed with 5 µg protein from whole extracts of HL cells in 20 µL shift buffer (2 µg poly(dI-dC), 1 µg BSA, 5 mM DTT, 20 mM HEPES (pH 8.4), 60 mM KCI and 8 % Ficoll) for 30 min at RT.

**ChIP.** ChIP assays were performed according to the Millipore protocol starting with 2 x 10^7^ cells/sample. For details see:

http://www.merckmillipore.com/DE/de/product/Chromatin-Immunoprecipitation-%28ChIP%29-Assay-Kit,MM_NF-17-295#anchor_MSD (download 20150715). Antibodies used were anti-RelA (Santa Cruz; sc-372, all replicates), anti-p50 (Santa Cruz; sc-1190, all replicates), anti-RelB (Santa Cruz; sc-226, all replicates), anti-p52 (Millipore #05-361, replicate 1 and #06-413, replicate 2). The RelA (sc-372) antibody was previously used in GSE19486 [4] and in GSE55105 [5]. The RelB (sc-226) and p50 (sc-1190) antibodies were also used in GSE55105 [5]. The p52 (Millipore #05-361) antibody specificity was verified by p52 ChIP with and without prior NFKB2 knockdown by siRNA. The p52 recruitment to selected ChIP-seq regions was reduced to a range of 6% to 17% compared to recruitment in cells treated with control siRNA (Figure S2D). For ChIP assays, cells were fixed with 1 % formaldehyde, lysed with 50 mM Tris-HCl, pH 8/ 5mM EDTA/ 1 % SDS and sonicated with the Bioruptor (Diagenode), using 12 cycles, setting M, sonication (30 s)/break (30 s) per cycle. Chromatin was pre-cleared with BSA saturated Protein A or G sepharose and incubated overnight at 4 °C with the appropriate antibody. Immuno-complexes were collected with BSA saturated Protein A or G sepharose for 1 h at 4 °C. Following washing, protein-DNA complexes were eluted using 1 % SDS/0.1 M NaHCO_3_. Reversion of cross-link, RNAse treatment, proteinase K digestion, and DNA purification with phenol/chloroform extraction were performed according to standard protocols. Quantitative PCR (qPCR) for validation of selected ChIP-seq regions was carried out with ChIP-DNA corresponding to 3 x 10^5^ cell equivalents using the CFX96 system and SsoFast EvaGreen Supermix (Bio-Rad Laboratories, München, Germany) in triplicates. Primer sequences are shown below. Non-recruiting regions from ACTB (actin, beta), POR (P450 (cytochrome) oxidoreductase), and a region downstream of the *NFKBIA* gene were used as references. A total of 4 ng input DNA was used as control. For quantification, the normalized expression (ΔΔCq) method was applied by using the CFX manager software (Bio-Rad) and the primer efficiencies are indicated in the following table.

**List of primers used for validation ChIP-seq regions**

| **Gene symbol and accession number** | **Primer name** | **Sequence (5' 🡪 3')** | **Primer position (TSS = 1)** | **Efficiency (%)** |
| --- | --- | --- | --- | --- |
| **AICDA**  NCBI36:12:8646029:8656706:-1 | AICDA_SP1 | GCAGCACAATTTCCAAGTCA | -16,609 | 96.0 |
|  | AICDA_RP1 | TGTCTCCCCTCACCAACTTC | -16,700 |  |
|  | AICDA_SP3 | TGGGATCAACCTGGATAGGA | 21,386 | 100.1 |
|  | AICDA_RP3 | CAGATTGCTCAACCCTCACA | 21,476 |  |
| **ATF3**  NCBI36:1:210805320:210860740:1 | ATF3_SP1 | TCTCAGTTTCACCCAAGTCC | 21,522 | 85.7 |
|  | ATF3_RP1 | TCGTCAGAGACAGAAAGTCC | 21,669 |  |
| **BCL2L1**  NCBI36:20:29715916:29774324:-1 | BCL2L1_SP3 | ACCATCACAGGGTTTTCCAG | 5,372 | 109.8 |
|  | BCL2L1_RP3 | CCGGGTAATTTTCGACAGAA | 5,457 |  |
| **BCL3**  NCBI36:19:49943644:49955141:1 | BCL3_SP1 | AAATCCCTTCCCGCAGAAC | 4,905 | 105.7 |
|  | BCL3_RP1 | GTTCCGGTAAAGTCTCAGCC | 5,047 |  |
|  | BCL3_SP2 | GGGACACAGAGACAGCAACA | -1,291 | 105.2 |
|  | BCL3_RP2 | AACTTGCCTGACCTCCCTCT | -1,210 |  |
| **BCL6**  NCBI36:3:188921859:188946169:-1 | BCL6_SP1 | GTGGGTGAGAGGTGGGACTA | 7,589 | 96.5 |
|  | BCL6_RP1 | CTTTCCCCTAAATCCCTCGT | 7,660 |  |
| **CCL22**  NCBI36:16:55950219:55957600:1 | CCL22_SP1 | GAGTGCCCCCTGTAAGAAAAC | -5,883 | 91.4 |
|  | CCL22_RP1 | GGACTGAAAGTGAGTGACAGAGAA | -5,808 |  |
| **CCR7**  NCBI36:17:35963550:35975250:-1 | CCR7_SP4 | CCAACATCTAGGTCAACCAC | 3,954 | 105.9 |
|  | CCR7_RP4 | GGCCTTGAGTCAGATCATCC | 4,079 |  |
| **CD70**  NCBI36:19:6536867:6542163:-1 | CD70_SP2 | CTCGCCAATTGCTCAAGTCC | -44 | 106.6 |
|  | CD70_RP2 | CGTCTACTTGCTTCAACCTG | 46 |  |
| **CSF2**  NCBI36:5:131437384:131439758:1 | CSF2_SP3 | CATCTTCAGCTGGATTCAACAC | 26,990 | 91.0 |
|  | CSF2_RP3 | AACTGATACCTTTCTGGGTGAC | 27,062 |  |
| **IL2RA**  NCBI36:10:6081835:6144278:-1 | IL2RA_SP3 | GCTGCATGCGTGACATCTCG | 11,025 | 82.7 |
|  | IL2RA_RP3 | AGGACCGGAAGCAGAGACCA | 11,139 |  |
| **JUNB**  NCBI36:19:12763310:12766124:1 | JUNB_SP4 | ATCTCCAGGGTTGGATGGTT | 1,992 | 103.8 |
|  | JUNB_RP4 | CCTGCGCACTCCAAGTCT | 2,084 |  |
| **NFKB2**  NCBI36:10:104143329:104152271:1 | NFKB2_SP3 | GAGTTAAACTTTCAGCCAAT | -169 | 88.0 |
|  | NFKB2_RP3 | CCGGGAAAGCCCCTTCT | -70 |  |
| **NFKBIA**  NCBI36:14:34940475:34943703:-1 | NFKBIA_SP1 | TTCAAATCGATCGTGGGAAAC | -352 | 101.3 |
|  | NFKBIA_RP1 | CAGCCTGCACCCTGTAATCC | -281 |  |
|  | NFKBIA_SP2 | CCCCAGCTCAGGGTTTAGG | -170 | 96.4 |
|  | NFKBIA_RP2 | CCAGTCAGACCAGAAAAAGAGAACT | -88 |  |
|  | NFKBIA_dist_SP1 | TGCCCAAGAAACATTCCCCA | 6,777 | 97.0 |
|  | NFKBIA_dist_RP1 | GGAGGGAAACACAGGTGAGG | 6,891 |  |
| **REL**  NCBI36:2:60962256:61003682:1 | REL_SP1 | CACTCGGAAGAACAACCTGG | -401 | 95.4 |
|  | REL_RP1 | ACGGCTAGCAGCGTGAGAAG | -333 |  |
| **RELB**  NCBI36:19:50196039:5023329:1 | RELB_SP3 | GCCAACCTCTCGATCCTGAA | -351 | 102.7 |
|  | RELB_RP3 | GCTTAGAGCCATCACGCCTT | -221 |  |
| **STAT5A**  NCBI36:17:37693091:37717484:1 | STAT5A_SP5 | TTTCCCTCTCAGGACTTCCA | 213 | 101.5 |
|  | STAT5A_RP5 | TCCGGCCATCTAAACTCAAC | 331 |  |
| **TP73**  NCBI36:1:3558989:3639716:1 | TP73_SP2 | GCATGTCTCCTGGTTGGTAA | 21,462 | 95.3 |
|  | TP73_RP2 | TCAGTTCACACTCAGCCAAG | 21,582 |  |
| **ACTB**  NCBI36:7:5533313:5536758:-1 | ACTB_SP1 | AGGCATCCTCACCCTGAAGTAC | 318 | 95.3 |
|  | ACTB_RP1 | TCTCCATGTCGTCCCAGTTG | 382 |  |
| **POR**  NCBI36:7:75381411:75454109:1 | POR_SP1 | CCTGAACCCACACAGTTCCT | 25,976 | 100.2 |
|  | POR_RP1 | CGAAGGACATGCTTCTGTCA | 26,066 |  |
|  | POR_SP2 | GCCAGAGCCTTGTCTAGGTG | 31,543 | 102.7 |
|  | POR_RP2 | GACACCCCAATCAAAGCAGT | 31,649 |  |

**ChIP-seq library construction.** Libraries were prepared using Illumina’s ChIP-Seq Sample Prep Kit (#IP-102-1001) according to the manufacturer’s instructions. Briefly, DNA was end-repaired using a combination of T4 DNA polymerase, *E. coli* DNA Pol I large fragment (Klenow polymerase), and T4 polynucleotide kinase. The phosphorylated blunt ends were treated with Klenow fragment (3' to 5' exo minus) and dATP to yield a protruding 3- ‘A’ base for ligation of Illumina's adapters which have a single ‘T’ base overhang at the 3’ end. After adapter ligation, size selection of the library was performed by excision of the region from 175 to 225 bp. DNA was PCR amplified with Illumina primers for 18 cycles. Each library was validated using an Agilent 2100 Bioanalyzer and sequenced on the Genome Analyzer II following the manufacturer’s protocols.

**Quality control of ChIP-seq libraries.** Total number of mapped reads (Reads), relative strand cross-correlation (RSC), normalized strand cross-correlation (NSC), and the ENCODE quality flag based on these two metrics (-2:veryLow,-1:Low,0:Medium,1:High,2:veryHigh). Input libraries are not expected to show high strand cross correlation.

| **Filename** | **Reads** | **NSC** | **RSC** | **QualityTag** |
| --- | --- | --- | --- | --- |
| Input_DNA_rep1_tech1.bam | 27.656.000 | 1.03 | 1.06 | 1 |
| Input_DNA_rep2_tech1.bam | 13.690.361 | 1.03 | 0.67 | 0 |
| Input_DNA_rep2_tech2.bam | 24.208.991 | 1.03 | 0.89 | 0 |
| Input_DNA_rep3_tech1.bam | 14.328.452 | 1.03 | 0.71 | 0 |
| Input_DNA_rep4_tech1.bam | 23.878.444 | 1.02 | 0.75 | 0 |
| Input_DNA_rep4_tech2.bam | 7.368.430 | 1.03 | 0.46 | -1 |
| Input_DNA_rep5_tech1.bam | 94.810.410 | 1.03 | 1.60 | 2 |
| Input_DNA_rep6_tech1.bam | 66.418.636 | 1.03 | 1.63 | 2 |
| p50_ChIPSeq_rep1_tech1.bam | 12.109.809 | 1.69 | 1.72 | 2 |
| p50_ChIPSeq_rep2_tech1.bam | 30.741.666 | 1.25 | 1.63 | 2 |
| p52_ChIPSeq_rep1_tech1.bam | 5.471.037 | 1.55 | 1.17 | 1 |
| p52_ChIPSeq_rep1_tech2.bam | 18.320.827 | 1.24 | 1.41 | 1 |
| p52_ChIPSeq_rep2_tech1.bam | 8.577.180 | 1.74 | 1.31 | 1 |
| p52_ChIPSeq_rep2_tech2.bam | 6.999.199 | 1.80 | 1.33 | 1 |
| RelA_ChIPSeq_rep1_tech1.bam | 11.433.500 | 1.06 | 0.95 | 0 |
| RelA_ChIPSeq_rep1_tech2.bam | 8.838.055 | 1.05 | 0.74 | 0 |
| RelA_ChIPSeq_rep2_tech1.bam | 14.933.215 | 1.12 | 2.24 | 2 |
| RelB_ChIPSeq_rep1_tech1.bam | 6.590.126 | 1.70 | 1.67 | 2 |
| RelB_ChIPSeq_rep2_tech1.bam | 7.303.234 | 1.74 | 2.70 | 2 |

**Peak calling and combinatorial analysis of NF-κB subunits.** Overlaps between peaks of different libraries were investigated as follows. First we defined the union of all regions bound in any of the experiments and then determined overlaps with peak calls from each of the individual libraries. Concerning the treatment of peaks in close proximity in one or more samples, we applied the following: (1) if none of the peaks overlap, they were treated independently no matter how close the proximity; (2) if two peaks in one sample were called independently in one sample, but overlap with the same peak in another sample, they were treated as a single larger peak.

To investigate combinatorial binding of subunits, ChIP-seq regions were classified according to their combinatorial binding pattern of NF-κB subunits into each of the 16 possible classes described by binary vectors of length four, where each of the four subunits is described by one digit. In addition, to avoid threshold effects of peak calling, we also applied κ-means clustering to the coverage profiles across all subunits. Coverage profiles were obtained for all regions with at least one peak call for any of the subunits. The regions of interest were defined as the average peak summit +- 500bp. Clustering was performed with the seqMINER tool [6] using enrichment over input, linear normalization, random seed 88268796, and κ = 8. Peaks were annotated to the closest gene and the closest transcription start site (TSS) within a window of 1 Mb according to the annotation of Ensembl version 54. Additional File 4: Table S2 contains the coordinates of the union of all regions along with gene assignments, information from the IDR analysis, and the results of the most significant MACS peak call in the region for each individual experiment as well as read counts in the region.

**Classification of the intergenic localization of ChIP-seq peaks.** The localization of ChIP-seq peaks has been classified based on the location of their summits: intergenic, 5’UTR, exon, intron, 3’UTR. The annotations of the hg18 mRNA transcriptome from UCSC that were downloaded contained 26,041 entries at the refGene table. The entries that aligned well against the 22 autosomes or the sexual chromosomes (25,542 entries) were filtered. Finally, the 24,756 mRNAs that had a unique genome alignment were selected. For each gene (18,342 different locus link ids), only one mRNA was chosen as representative, with the most 5’ TSS. In case of several transcript variants, the longest was selected. ChIP-seq regions (summit) that overlapped with more than one gene (54 ChIP-seq regions for p50 and 51 for p52) were not considered.

**Motif search.** For each ChIP-seq dataset of the NF-κB subunits motif analyses were performed on either the full dataset or on specific subsets (not overlapping ChIP-seq regions of p50 and p52). Each motif analysis used a maximal number of 5,000 randomly selected ChIP-seq regions having a maximal length of 1,200 nucleotides. For the *de novo* motif search, the parallel version of the Multiple EM for Motif Elicitation (MEME) motif discovery tool (version 4.6.1) was used with 500 CPU cores on a linux-based computer cluster and the following command line parameters: Maximal number of returned motifs equaled 20 (nmotifs), minimal motif size equaled 7 nucleotides (minw), maximal motif size equaled 15 nucleotides (maxw), reverse complement search was activated (revcomp), minimal number of sites equaled 10 % of the dataset size in sequences (minsites), maximal number of sites equaled 100% of the dataset size in sequences (maxsites), and the E-value threshold (evt) equaled 0.0001. To search for known motifs, 76 human motifs from the JASPAR CORE database (downloaded on 28 February 2011) and the CLOVER motif search tool [7], compiled on 13 November 2009, were used. Motifs were selected if significantly enriched (*P* <0.01) in a ChIP-seq dataset in comparison to several background sets provided with the tool (that is, mononucleotide randomization, dinucleotide randomization, sequences 2000 bp upstream of human genes, human chromosome 20).

**RNA isolation and Affymetrix gene chip processing.** Total RNA from L1236 cells subjected to RNAi treatments was prepared following manufacturer’s protocol (RNeasy Kit; QIAGEN, Hilden, Germany). RNA concentrations were measured using a NanoDrop ND-1000 UV-visible spectrophotometer (Nanodrop Technologies). RNA quality was assessed by 260/280 and 260/230 ratios and additionally by RNA Integrity Number (RIN) using eukaryote total RNA nano chip in the Agilent 2100 Bioanalyser (Agilent Technologies). Only RNA samples with 260/280 and 260/230 ratios higher than 1.8, and RIN higher than 8.5, were used for preparation of microarray samples. Microarray experiments were carried out following Ambion (Ambion, AMB), and Affymetrix (Affymetrix Inc, Santa Clara, CA, USA) protocols. Samples were prepared from 100 ng of total RNA, which were reverse transcribed to cDNA followed by *in vitro* transcription, fragmentation of the single stranded cDNA and labeling. The biotinylated cDNAs were hybridized to Affymetrix GeneChip Human Gene 1.0 ST Arrays (Affymetrix Inc, Santa Clara, CA, USA).

**Analysis of gene expression data.** Data were normalized using Robust Multi-Array Average (RMA) with background correction and quantile normalization as implemented in the Bioconductor library ‘Oligo’. The log transformed expression levels of each Affymetrix transcript cluster were adjusted for available covariates by adding the residuals from a linear model using all covariates as predictors to the mean expression level. Genes with low expression values (Affymetrix hybridization value <6) in half of the samples were removed from the analysis. In order to obtain unambiguous assignments of transcript clusters to Ensembl genes, the annotation from the Bioconductor package hugene10sttranscriptcluster.db was used. All genes that were located on chromosomes other than 1-22, X, Y, and MT according to Ensembl version 54 (NCBI36/hg18 genome build) were removed. Because of their high sequence similarity, genes from the histone gene clusters were also removed.

**Definition of specific transcription factor target genes.** For each ChIP-seq experiment, transcription factor (TF) binding was summarized at the gene level. A gene was defined as bound if it was assigned to either the closest gene or the closest TSS of at least one peak. A gene was called differentially expressed if at least one transcript cluster assigned to the gene was called differential (FDR <0.05) and showed at least 10 % expression difference between the KD and control experiment. The target genes of a specific TF were defined as the overlap of differentially expressed genes upon knockdown (KD) and genes bound by the TF. The significance of this overlap was assessed by Fisher’s exact test, considering the set of genes used for differential expression analysis as the reference set. For each target the mode of regulation was determined according to the expression change as activated (downregulated after the KD of the TF) or repressed (upregulated after the KD of the TF). Targets of the canonical NF-κB pathway were defined by integrating the differential expression results of the double KD of *NFKB1* and *RELA* with the ChIP-seq results for p50, while the targets of the non-canonical NF-κB pathway were defined by integrating the differential expression results of the double KD of *NFKB2* and *RELB* with the ChIP-seq results for p52. For the comparison of the canonical and non-canonical target gene sets the classification of genes was further refined whether they were regulated in both cases (common) or exclusively in one and whether they were activated or repressed. On each of the sets functional enrichment analysis was performed using the biological process gene ontology (GO). The *P* values from the hypergeometric tests were adjusted for testing multiple sets for multiple GO terms using the Benjamini–Hochberg method.

**Reverse transcription quantitative PCR (RT-qPCR).** One microgram of RNA from the samples prepared for Affymetrix GeneChip processing was reverse transcribed using the iScript™cDNA Synthesis Kit (Bio-Rad). The quantitative PCR was carried out with pooled, 20-fold diluted cDNA from three biological replicates using the CFX96 system and SsoFast EvaGreen Supermix (Bio-Rad) with triplicate reads. Primer sequences are listed below. *HPRT1* (hypoxanthine phosphoribosyltransferase 1), *RPL13A* (ribosomal protein L13a), and *TBP* (TATA box binding protein) were used as references with a mean M value of 0.12. cDNA from cells treated with control siRNA in the respective KD experiment was used as control. For quantification, the normalized expression (ΔΔCq) method was applied by using the CFX manager software (Bio-Rad) and the primer efficiencies indicated in the listing below.

**List of primers used in RT-qPCR experiments**

| **Gene symbol and accession number** | **Primer name** | **Sequence (5'→3')** | **Position (TSS=1)** | **Efficiency (%)** |
| --- | --- | --- | --- | --- |
| **HPRT1**  NM_000194.2 | hHPRT1_RT_SP3 | TGTAATGACCAGTCAACAGGG | 483 | 101.5 |
|  | hHPRT1_RT_RP3 | GGATTATACTGCCTGACCAAGG | 631 |  |
| **RPL13A**  NM_012423.2 | hRPL13A_RT_SP2 | AAAGCCAAGATCCACTACCG | 509 | 105.7 |
|  | hRPL13A_RT_RP2 | GGAATTAACAGTCTTTATTGGGCTC | 657 |  |
| **TBP**  NM_003194.4 | hTBP_RT_SP2 | GGGTTTTCCAGCTAAGTTCTTG | 1,011 | 100.8 |
|  | hTBP_RT_RP2 | CTGTAGATTAAACCAGGAAATAACTCTG | 1,060 |  |
| **NFKB2**  NM_002502.3 | NFKB2_RT_SP7 | CCGGATCTCGCTCTCCACCGGA | 65 | 86.2 |
|  | NFKB2_RT_RP7 | GGGCTAGGCCCGGCTCTGTCTA | 188 |  |
| **NFKB1**  NM_003998.3 | NFKB1_RT_SP2 | CATCCACCTTCATTCTCAACTTG | 2,123 | 100.0 |
|  | NFKB1_RT_RP2 | ACCACATCTTCCTGCTTAGTG | 2,263 |  |
| **RELB**  NM_006509.3 | RELB_RT_SP1 | CTACAACGCTGGGTCCCTGA | 878 | 103.9 |
|  | RELB_RT_RP1 | TAGACGGGCTCGGAAAGCAC | 1,009 |  |
| **RELA**  NM_021975.3 | RELA_RT_SP1 | CCTGTCCTTTCTCATCCCATC | 668 | 87.3 |
|  | RELA_RT_RP1 | ACCTCAATGTCCTCTTTCTGC | 816 |  |
| **CCL22**  NM_002990.4 | CCL22_RT_SP1 | GAAACACTTCTACTGGACCTC | 184 | 84.5 |
|  | CCL22_RT_RP1 | GCACAGATCTCCTTATCCCT | 264 |  |
| **IER3**  NM_003897.3 | IER3_RT_SP1 | AGTCGCCTTTAGGGTGGCTG | 1,122 | 97,4 |
|  | IER3_RT_RP1 | ATCTCGACAGTCGCTCCGTG | 1,199 |  |
| **TNFAIP3**  NM_001270507.1 | TNFAIP3_RT_SP1 | TCCTGCCTTGACCAGGACTTG | 254 | 100.3 |
|  | TNFAIP3_RT_RP1 | CATTGTGCTCTCCAACACCTCT | 349 |  |
| **NFKBIA**  NM_020529.2 | NFKBIA_RT_SP1 | GAGGACGAGCTGCCCTATGA | 1,008 | 98.9 |
|  | NFKBIA_RT_RP1 | AGCCCCTTTGCGCTCATAAC | 1,077 |  |
| **CD44**  NM_000610.3 | CD44_RT_SP2 | TACATCCTCACATCCAACACC | 747 | 97,9 |
|  | CD44_RT_RP2 | GTGCCATCACGGTTAACAATAG | 892 |  |
| **CFLAR**  NM_003879.5 | CFLAR_RT_SP1 | CCTCACCTTGTTTCGGACTATAG | 724 | 100.8 |
|  | CFLAR_RT_RP1 | TCCTTGCTTATCTTGCCTCG | 848 |  |
| STAT5A  NM_003152.3 | STAT5A_RT_SP4 | GCTCCCTCTCATGAATGTTTG | 3,014 | 111.7 |
|  | STAT5A_RT_RP4 | ACAGCCTTACTAAACTCACAAC | 3,103 |  |
| CD40  NM_001250.4 | CD40_RT_SP1 | GAGAGCCCTGGTGGTGATCC | 653 | 96.8 |
|  | CD40_RT_RP1 | GGCTTCTTGGCCACCTTTTTGAT | 742 |  |
| FOXP1  NM_032682.5 | FOXP1_RT_SP3 | ACCTGCATGTGAAGTCTACAG | 1,629 | 90,8 |
|  | FOXP1_RT_RP3 | GGTCGTTGGAGTATGAGGTAAG | 1,752 |  |
| PTK2  NM_005607.4 | PTK2_RT_SP1 | ACAGCTACAACGAGGGTGTCA | 2,986 | 87.4 |
|  | PTK2_RT_RP1 | CGGTCCAGGTTGGCAGTAGG | 3,055 |  |
| BCL6  NM_001706.4 | BCL6_RT_SP1 | AATTTTGGACTGTGAAGCAAGG | 328 | 93.5 |
|  | BCL6_RT_RP1 | GGCGGGTGAACTGGATAC | 405 |  |

**Effects of combinatorial binding of NF-κB subunits on gene expression.** The goal of this analysis was to predict the consequences of combinatorial binding of NF-κB subunits on expression. Towards this end we have setup a framework consisting of four components and systematically evaluated its prediction performance for different parameter choices. The four components are: (1) filtering of binding regions, (2) classification of binding regions, (3) assignment of regions to genes, (4) gene level aggregation. We considered the following options for each component.

(1) *Filtering of binding regions*: We considered (i) filtering out regions that do not overlap with DHS or (ii) to perform no filtering.

(2) *Classification of binding regions:* (i) We classified regions based on their combinatorial binding pattern of NF-κB subunits into each of the 16 possible classes described by binary vectors of length four, where each of the four subunits is described by one digit. (ii) We used the cluster assignment obtained from k-means clustering of the occupancy profiles (Figure 2C).

(3) *Assignment of regions to genes:* We considered different assignment methods based on the distance between regions and gene annotations, as well as methods that made use of Hi-C data measured in GM12878 [8]. For the distance-based assignments we selected for each region (i) the closest gene, (ii) the closest TSS, or (iii) any of the two. The contact matrix of the Hi-C experiment was downloaded from the GEO database [9] using the accession number GSE63525. We selected all contacts that were overlapping with our ChIP-seq regions on the one side and with TSS annotations (+-2kb) on the other side. For each pair of ChIP region and TSS we counted the number of contacts. Since there is a strong dependence of the number of contacts on the distance between the two regions, we fitted a generalized linear model ‘contacts ~ log (distance)’ using the negative binomial family and the log as link function as a background model. From this model we obtained *P* values for the number of contacts exceeding the expected numbers and the corresponding FDR. Finally, we considered assignments with at least five contacts for each region (iv) choosing the gene with the highest number of contacts, (v) all genes with at least five contacts, (vi) all genes with contacts that have FDR <5 %, (vii) all genes with contacts that have FDR <5 % or are closer than 10 kb, (viii) for each region choosing the gene with the smallest *P* value.

(4) *Gene level aggregation*: (i) We counted how many regions of each class were assigned to each gene. (ii) We transformed these counts to binary variables indicating counts greater zero. In addition we also considered counting and indicators for proximal (distance less than 10 kb) and distal regions separately.

Each gene was then classified according to its expression pattern upon the KD of the canonical (*NFKB1* + *RELA*) and non-canonical (*NFKB2* + *RELB*) dimers into activated or repressed genes based on the direction of the fold change. Moreover we also classified genes into genes regulated by the canonical or non-canonical dimers based on the test for differential gene expression (FDR <0.05).

First, we searched for binding patterns predictive of activation versus repression. In particular, we considered all genes that were significantly differentially expressed in at least one KD experiment and assigned them to the classes ‘activated by both pathways’ or ‘repressed by both pathways’. Second, we searched for binding patterns predictive of regulation by the canonical pathway, the non-canonical pathway, or no regulation at all. In particular we defined the following binary classifications of genes: ‘exclusively canonical’ versus ‘exclusively non-canonical’, ‘canonical’ versus ‘non-regulated’, and ‘non-canonical’ versus ‘non-regulated’. For each of the binary classification problems we fitted a logistic regression model

with the counts or indicators x_ij_ of binding regions of each region class for each gene i as the predictor variables, where P indicates the set of possible region classes. The prediction performance was evaluated using 10-fold cross-validation. As a measure we used the average across the 10-folds of the area under the receiver operator characteristics curve (AUCROC). We performed a systematic evaluation for all of the classification tasks and all combinations of the four components of the framework described above. Finally, we selected the combination of components that yielded the best performance averaged over the classification tasks. The best set-up was (1) no filtering of regions for overlap with DHS, (2) region classification according to subunit binding patterns, (3) assignment to the closest gene or closest TSS, (4) gene level aggregation by counting regions separately by distance (Additional File 2: Figure S5). Using these settings, we additionally compared ‘activated by both pathways’ versus ‘non-regulated’, and ‘repressed by both pathways’ versus ‘non-regulated’. We performed a bootstrapped feature selection to robustly identify which binding patterns were predictive in each classification task. We drew N = 100 bootstrap samples from our data, such that the number of data points assigned to each class was preserved. For each bootstrap sample, we estimated the model parameters and tested each of the hypotheses β_j_ = 0 using the likelihood ratio test. Finally, we counted for each β_j_ how many times it was selected as significant feature (*P* <0.01) across the bootstrap samples to obtain a feature confidence score.

**Comparison with DNAse I hypersensitive regions.** We obtained the 25,865 processed DNAse hypersensitive sites (DHS) for the L1236 cell type defined in [10] from the gene expression omnibus (GEO) database [9] under accession number GSM1251561. For comparison we also obtained DHS for the GM12878 cell type from the ENCODE website http://hgdownload.cse.ucsc.edu/goldenPath/hg18/encodeDCC/wgEncodeChromatinMap/wgEncodeDukeDNaseSeqPeaksGm12878V3.narrowPeak.gz. In addition, to account for the large difference in the number of DHS calls between cell types, we also provide a comparison using only the top 25,865 sites ranked by their *P* values. These results are referred to as ‘matched’ in Additional File 7: Table S5. For all of our binding regions we tested whether or not they overlap by at least 1 bp with the DHS regions from L1236 or GM12878.

**Comparison with NF-κB subunit binding in lymphoblastoid cell types.** NF-κB subunit binding was previously characterized in the GM12878 cell type [5]. The authors of this study were so kind to provide us with the processed subunit peak calls. We transferred coordinates from hg19 to hg18 using the UCSC liftover tool [11]. For the comparison of subunit binding patterns and DHS regions between cell types we proceeded as described in the section ‘Analysis of ChIP-seq data’, counting overlaps based on the union of all regions. For each subunit we computed the percentage of overlap relative to the total number of regions bound by the respective subunit in each of the two cell types. We determined the percentage of regions bound by the same subunit in both cell types (labeled ‘ChIP in ChIP’ in Additional File 6: Table S4). In addition we characterized the overlap with DHS in each cell type separately (labeled ‘ChIP in DHS’) and with DHS present in any of the two cell types (labeled ‘ChIP in any DHS’). Moreover, we also characterized the overlap with DHS for regions that were bound specifically only in L1236. For each subunit we indicated whether regions called by us in L1236 overlap by at least 1bp in Additional File 7: Table S5. To assess the degree to which distinct combinations of subunits are conserved between cell types, we classified these regions according to their combinatorial binding pattern of NF-κB subunits (see section ‘Analysis of ChIP-seq data’) also in the GM12878 cell type. Finally, we determined the percentage of regions with identical binding patterns in the other cell type for each of the cell types (Additional File 7: Table 5).

**Alamar Blue assay.** Cell viability was monitored using the Alamar Blue assay (Invitrogen, Camarillo, CA, USA). L1236 cells were incubated with two distinct siRNA sequences against *NFKB1*+*RELA*, *NFKB2*+*RELB*, *BCL2L1*, or siRNA non-targeting sequences for 3 days and normal cultivation conditions were then re-established for 3 additional days. After the treatment period, the standard medium was replaced with medium containing 10 % Alamar Blue dye and the samples were incubated for 6 h in a 37 °C humidified incubator with 5 % CO2. Absorbance was then measured at 570 nm, using 600 nm as a reference wavelength (normalized to the 600 nm value) on a spectrophotometer. Tests were performed in triplicate.

**Trypan Blue exclusion test of cell viability.** After treating HL cells with specific siRNAs (see figure legends), time-course experiments were performed using a 0.4 % Trypan Blue solution (Sigma) to determine the viability of cells. The number of viable cells was defined by subtraction of the number of cells that were stained with Trypan Blue from the total number of cells. To calculate the relative percentages of viable cells, the number of cells in the siRNA-treated samples was normalized with the number of cells in the non-treated control sample. Experiments were performed in triplicates.

**Integration of NF-κB signatures with gene expression in human lymphomas.** Probesets obtained from GEO (GSE12453) were assigned to Ensembl genes using the Bioconductor package hgu133plus2. Target genes in HL were defined as the overlap of genes that were defined as transcription factor targets above with genes that had at least one differentially expressed probeset (FDR <0.05) comparing HL samples to the rest. Significance of the overlap was determined using Fisher’s exact test with all genes that were considered in any of the differential expression analyses as the reference set. Genes with concordant expression changes, that is, activation by the transcription factor and upregulation in HL cells and vice versa, were selected for visualization.

**Supplemental references**

1. Schreiber E, Matthias P, Muller MM, Schaffner W. Rapid detection of octamer binding proteins with 'mini-extracts', prepared from a small number of cells. Nucleic Acids Res. 1989;17(15):6419.

2. Hinz M, Stilmann M, Arslan SC, Khanna KK, Dittmar G, Scheidereit C. A cytoplasmic ATM-TRAF6-cIAP1 module links nuclear DNA damage signaling to ubiquitin-mediated NF-kappaB activation. Mol Cell. 2010;40(1):63–74.

3. Naumann M, Wulczyn FG, Scheidereit C. The NF-kappa B precursor p105 and the proto-oncogene product Bcl-3 are I kappa B molecules and control nuclear translocation of NF-kappa B. EMBO J. 1993;12(1):213–222.

4. Kasowski M, Grubert F, Heffelfinger C, Hariharan M, Asabere A, Waszak SM, et al. Variation in transcription factor binding among humans. Science. 2010;328(5975):232–235.

5. Zhao B, Barrera LA, Ersing I, Willox B, Schmidt SC, Greenfeld H, et al. The NF-kappaB genomic landscape in lymphoblastoid B cells. Cell Rep. 2014;8(5):1595–1606.

6. Ye T, Krebs AR, Choukrallah MA, Keime C, Plewniak F, Davidson I, et al. seqMINER: an integrated ChIP-seq data interpretation platform. Nucleic Acids Res. 2011;39(6):e35.

7. Frith MC, Fu Y, Yu L, Chen JF, Hansen U, Weng Z. Detection of functional DNA motifs via statistical over-representation. Nucleic Acids Res. 2004;32(4):1372–1381.

8. Rao SS, Huntley MH, Durand NC, Stamenova EK, Bochkov ID, Robinson JT, et al. A 3D map of the human genome at kilobase resolution reveals principles of chromatin looping. Cell. 2014;159(7):1665–1680.

9. Barrett T, Wilhite SE, Ledoux P, Evangelista C, Kim IF, Tomashevsky M, et al. NCBI GEO: archive for functional genomics data sets--update. Nucleic Acids Res. 2013;41(Database issue):D991–995.

10. Kreher S, Bouhlel MA, Cauchy P, Lamprecht B, Li S, Grau M, Hummel F, et al. Mapping of transcription factor motifs in active chromatin identifies IRF5 as key regulator in classical Hodgkin lymphoma. Proc Natl Acad Sci U S A. 2014;111(42):E4513–4522.

11. Hinrichs AS, Karolchik D, Baertsch R, Barber GP, Bejerano G, Clawson H, et al. The UCSC Genome Browser Database: update 2006. Nucleic Acids Res. 2006;34(Database issue):D590–598.
