# Supplementary material for: A roadmap of constitutive NF-κB activity in Hodgkin lymphoma: Dominant roles of p50 and p52 revealed by genome-wide analyses
Source: Genome Med. 2016 Mar 17;8:28. doi: 10.1186/s13073-016-0280-5 (PMC4794921; doi:10.1186/s13073-016-0280-5)
Supplement: Additional file 2: Figure S1 — shows co-immunoprecipitation of p50 and p52 with NF-κB subunits. Figure S2 shows the number of ChIP-seq regions bound by the individual NF-κB subunits, the number of genes assigned to ChIP-seq regions, the validation of ChIP-seq regions by qPCR, the frequency of ChIP-seq regions for NF-κB subunits relative to the transcription start site (TSS), p52 recruitment to selected ChIP-seq regions upon treatment of L1236 cells with NFKB2-siRNAs and control-siRNAs, and the genomic locations of p50 and p52-ChIP-seq regions with respect to annotated genes. Figure S3 shows p50- and p52-selective motifs detected by using MEME. Figure S4 shows the optimization and specificity controls for gene knockdown experiments. Figure S5 shows the prediction of gene expression patterns from combinatorial binding of NF-κB subunits. Figure S6 shows cell death induction by depletion of p50/RelA, p52/RelB or Bcl-XL. Figure S7 shows RT-qPCR analyses of genes that were differentially expressed in classical HL compared to other lymphoid malignancies and normal B cells. (PDF 3236 kb) [file 13073_2016_280_MOESM2_ESM.pdf]

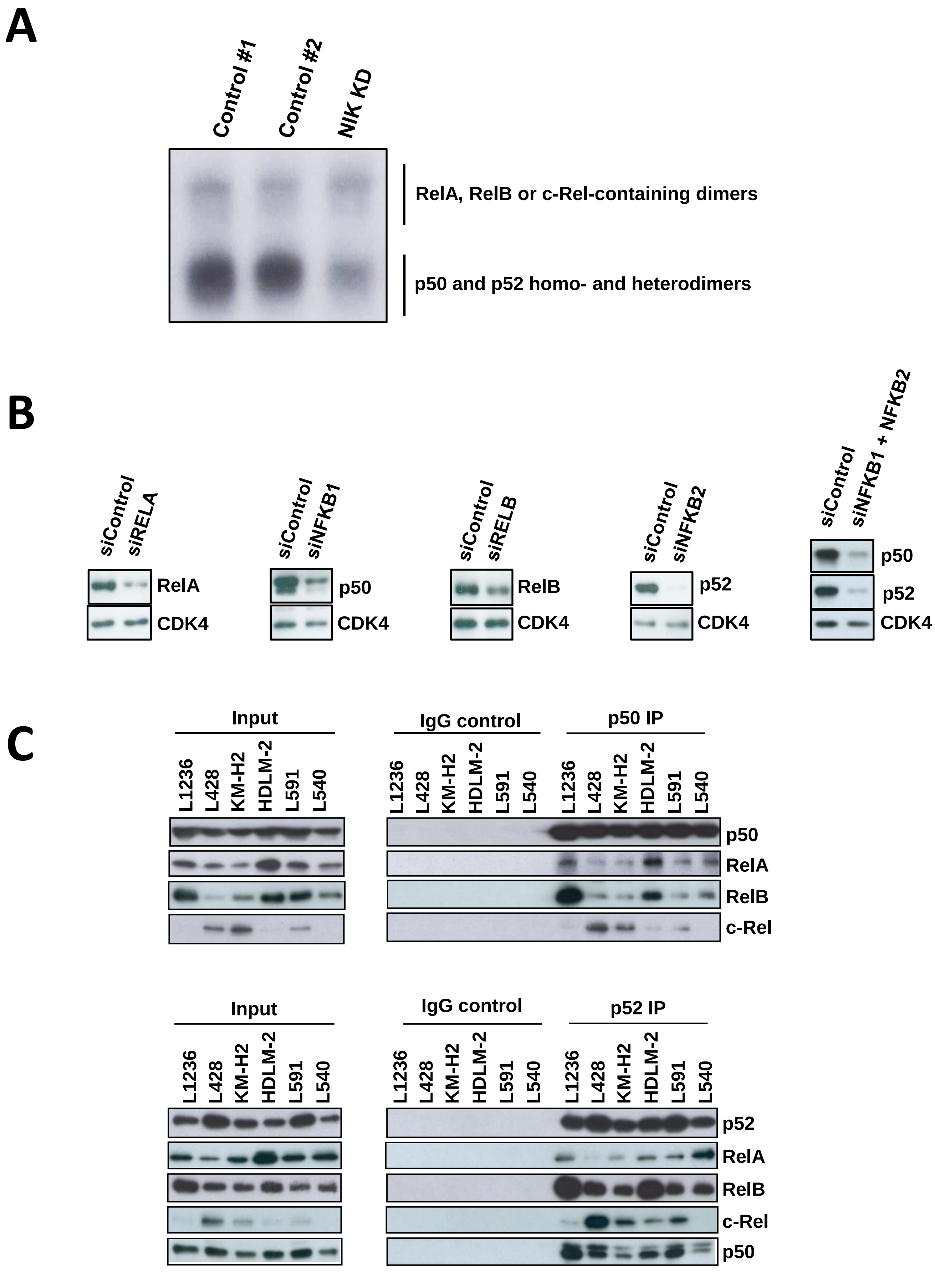
A
Control #1
Control #2
NIK KD
RelA, RelB or c-Rel-containing dimers
p50 and p52 homo- and heterodimers
B
siControl
siRELA
RelA
CDK4
siControl
siNFKB1
p50
CDK4
siControl
siRELB
RelB
CDK4
siControl
siNFKB2
p52
CDK4
siControl
siNFKB1 + NFKB2
p50
p52
CDK4
C
Input
IgG control
p50 IP
L1236
L428
KM-H2
HDLM-2
L591
L540
p50
RelA
RelB
c-Rel
Input
IgG control
p52 IP
L1236
L428
KM-H2
HDLM-2
L591
L540
p52
RelA
RelB
c-Rel
p50

Figure S1 (related to Figure 1). Contribution of p50 and p52 to constitutive NF-κB activity in HL cells. (A) EMSA analysis of NF-κB activity in whole extracts from L1236 cells treated with siControl (Control #1), siRIPK1 (Control #2) and siMAP3K14 (NIK KD) and harvested 1 day after the end of the siRNA treatment. (B) WB showing the KD efficiencies of NF-κB subunits in whole cell extracts used for EMSA assay in Figure 1A. (C) Co-immunoprecipitation (IP) of p50 and p52 with NF-κB subunits. Antibodies against p50 (top panel) and p52 (bottom panel) were used to IP the respective proteins from nuclear extracts of HL cells. Western blot was used to detect other NF-κB subunits, as indicated. Input levels of proteins are shown in the left panels.

A

Recruited Protein	ChIP-seq Regions	Assigned Genes
p50	10,975	7,328
p52	10,893	6,556
RelA	492	462
RelB	3,984	3,224

B

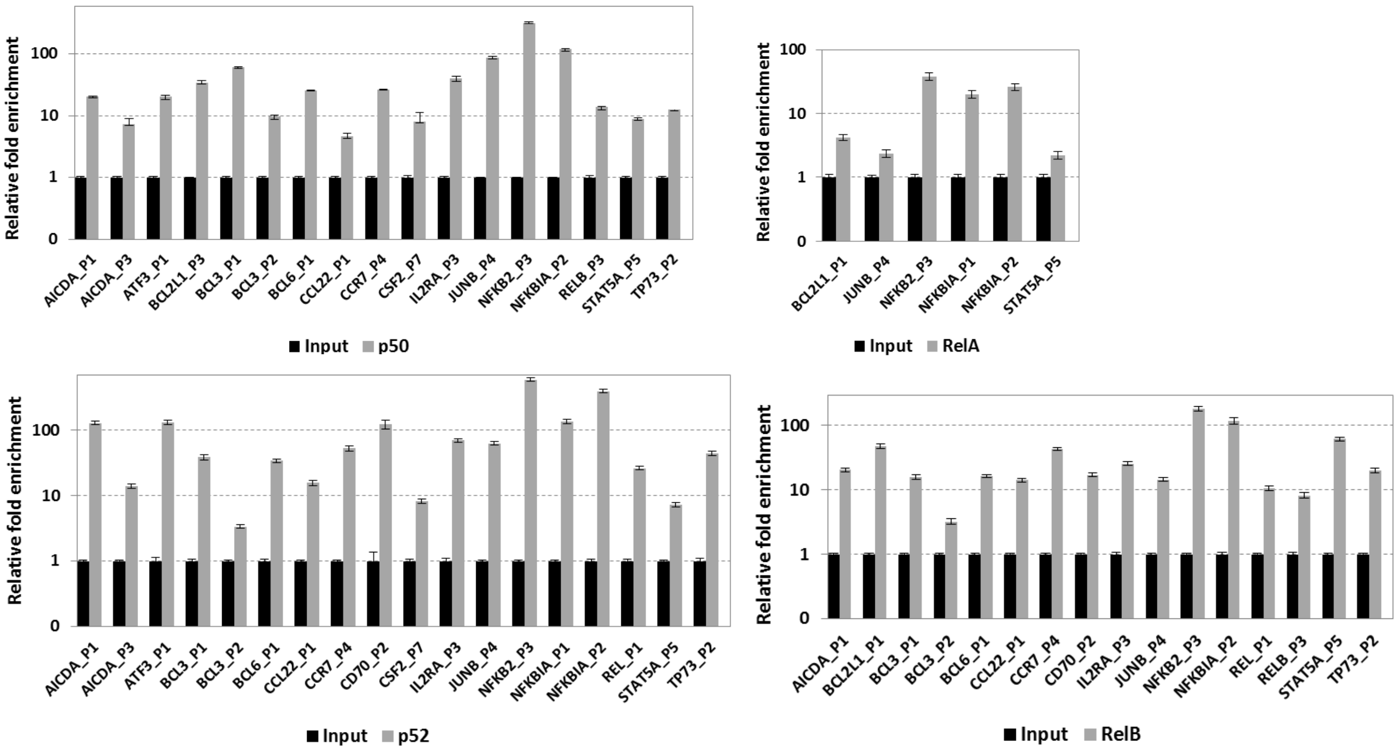

C

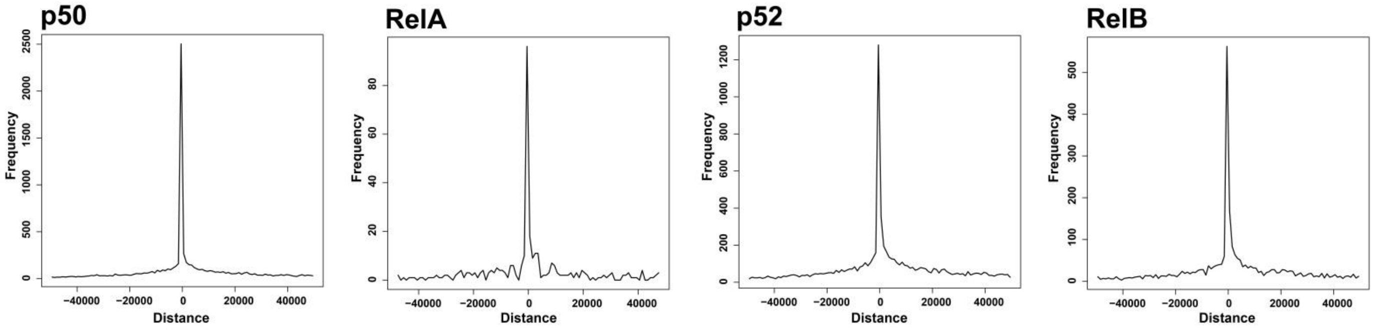

Figure S2 (related to Figure 2)

D

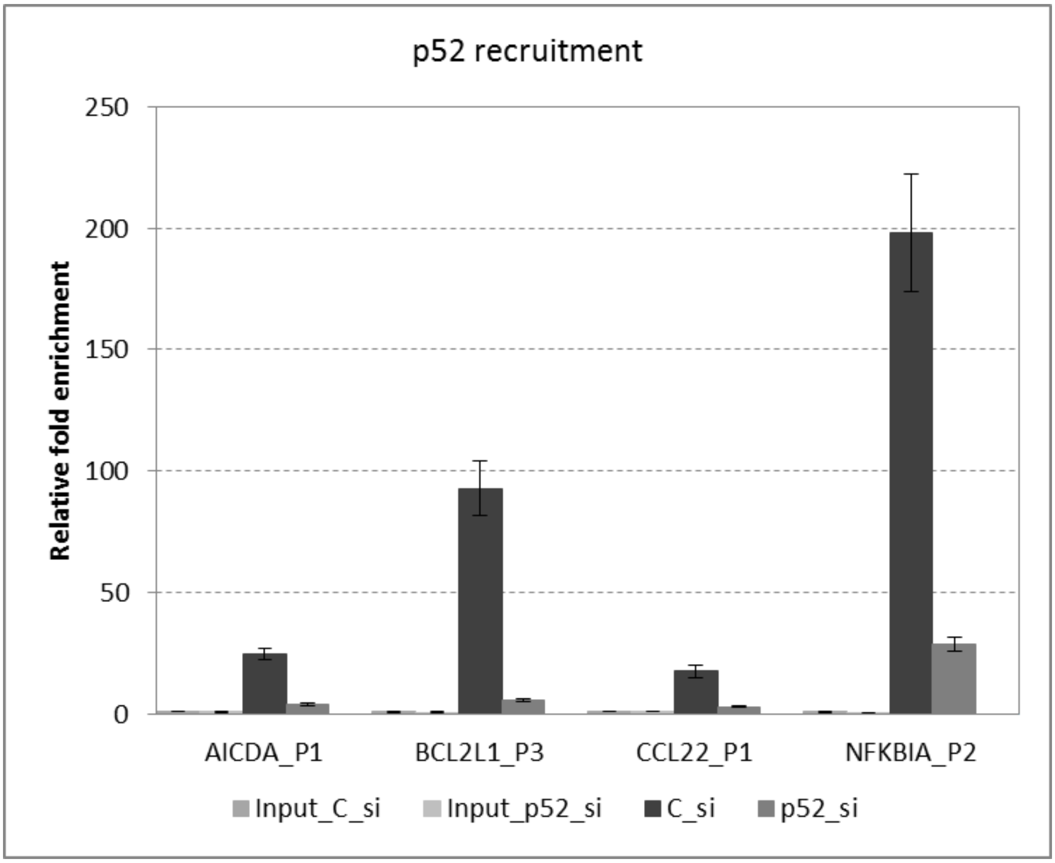

E

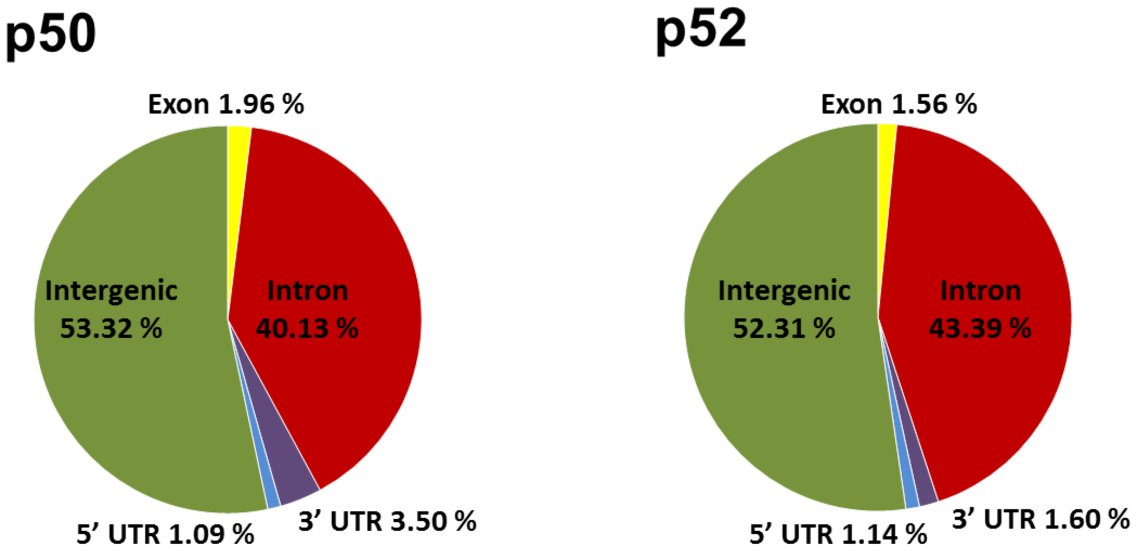

Continuation of Figure S2 (related to Figure 2)

Figure S2 (related to Figure 2). Analysis of genome-wide NF-κB subunit recruitment. (A) Number of ChIP-seq regions bound by the individual NF-κB subunits in L1236 cells and number of genes assigned to ChIP-seq regions. (B) Validation of ChIP-seq regions by qPCR. Results are shown as relative fold enrichment over the input with three reference regions devoid of NF-κB binding. Error bar = SEM of triplicate reads. (C) Frequency of ChIP-seq regions for NF-κB subunits relative to the transcription start site (TSS). Numbers on the x-axis denote positions relative to the TSS. (D) p52 recruitment to selected ChIP-seq regions upon treatment of L1236 cells with NFKB2-si and Control-si, respectively. (E) Genomic locations of p50 and p52–ChIP-seq regions with respect to annotated genes.

Continuation of Figure S2 **(related to Figure 2)**

p50

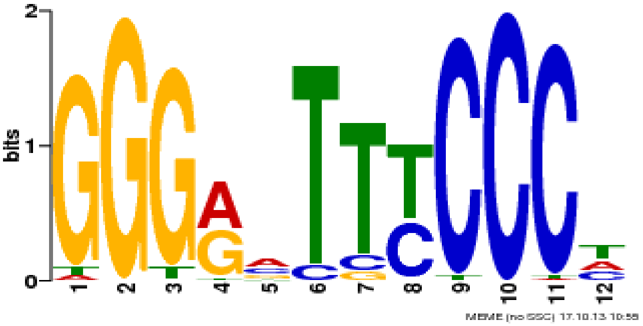

p52

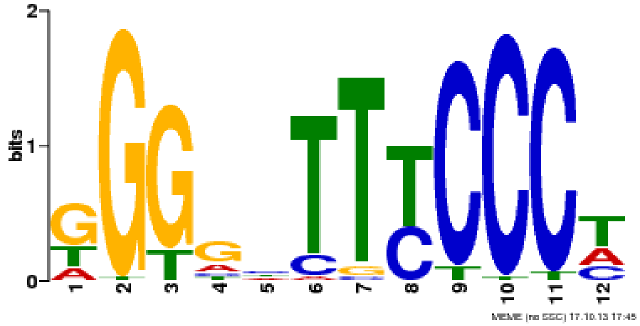

Figure S3 (related to Figure 3). p50- and p52-selective motifs. Motifs detected by *de novo* search, using MEME, in ChIP-seq regions that are selectively enriched for either p50 (top) or p52 (bottom).

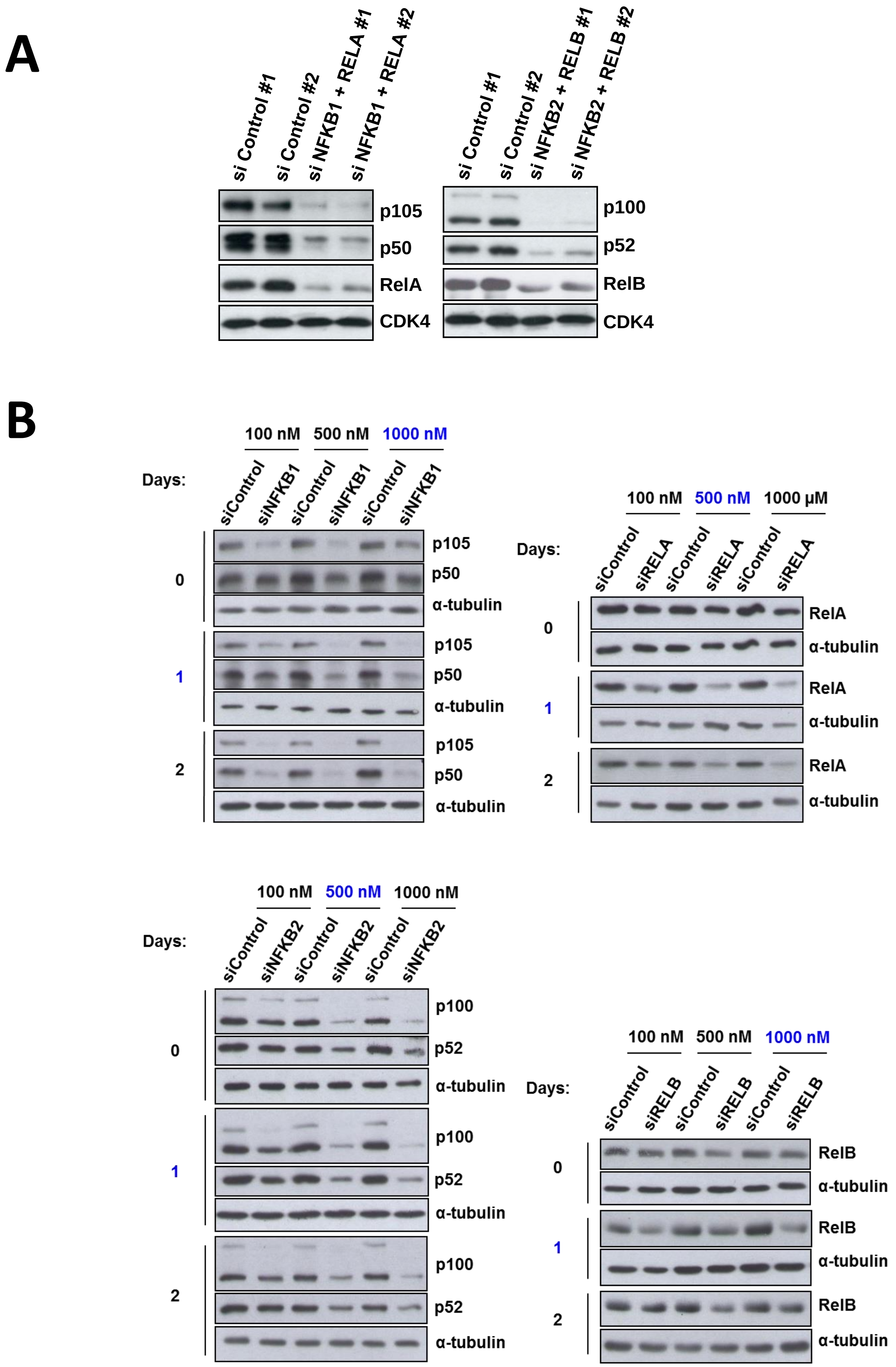

Figure S4 (related to Figure 4)

C

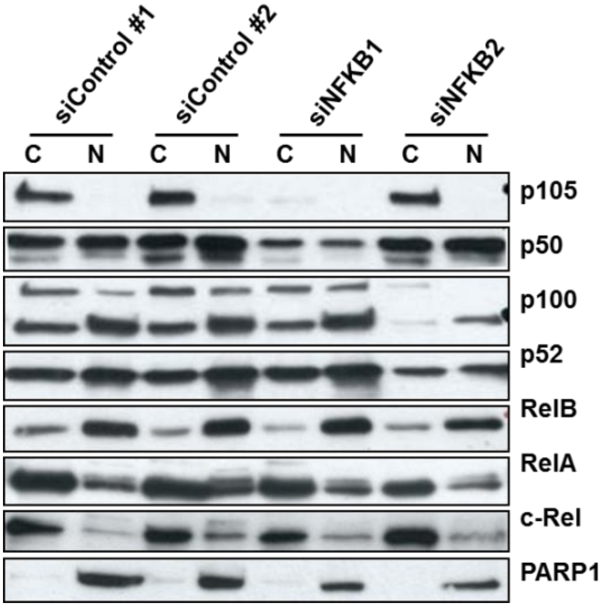

Figure S4 (related to Figure 4). Optimization and specificity controls for gene knockdown experiments. (A) The depletion of NF-κB subunits by siRNA treatment in L1236 cells was visualized by Western blotting. Two distinct siRNAs for each NF-κB subunit and two non-targeting siRNAs were used. (B) Titration of siRNA concentrations for each NF-κB subunit and determination of optimal siRNA incubation times. The concentrations and incubation times chosen for transcriptome analyses are highlighted in blue. Time 0 is the time point of the end of the siRNA treatment and the start of the re-establishment of the normal FBS conditions (siRELA and siNFKB2 = 2 days; siRELB and siNFKB1 = 3 days). α-Tubulin was used as loading control. (C) p105/p50 and p100/p52 KD was followed by cell fragmentation and protein level analyses of NF-κB subunits by WB. PARP1 and p105 serve as purity controls of cytoplasmic and nuclear extracts, respectively.

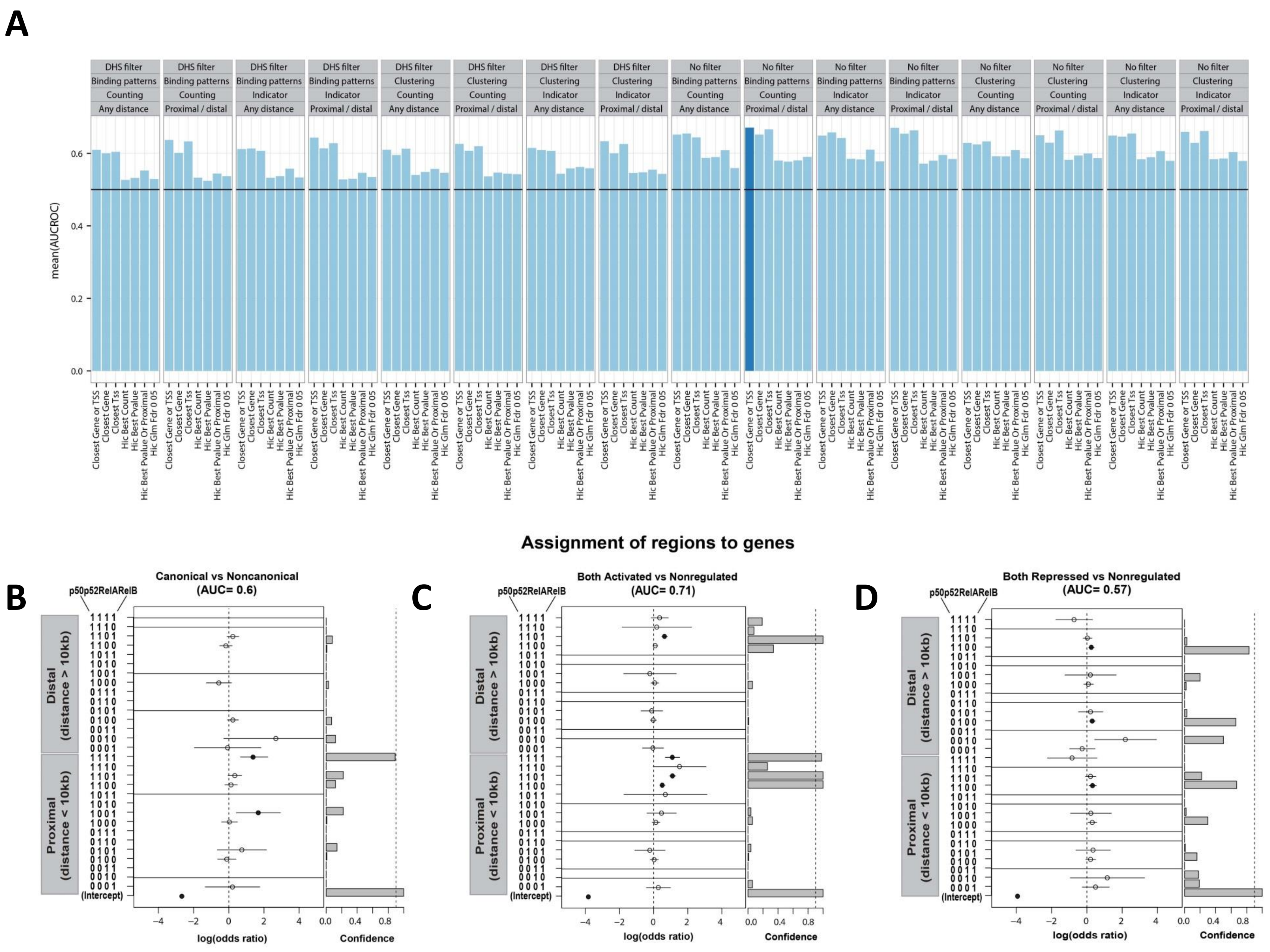

Figure S5 (related to Figure 5)

Figure S5 (related to Figure 5). Prediction of gene expression patterns from combinatorial binding of NF-κB subunits. Application of logistic regression models to predict gene expression patterns from combinatorial binding of NF-κB subunits. The performance of the prediction framework for the different parameter choices was systematically evaluated (see Supplementary Procedures). The best combination of parameters was selected according to the average AUCROC across all prediction tasks shown dark blue in panel (A). The grey top panels in each subfigure show which parameter settings were used for filtering, region classification, gene level aggregation and whether distances between regions and genes were considered or not (from top to bottom). Region to gene assignments are shown on the x-axis. Using these optimized parameters, bootstrapped feature selection was performed to identify the most predictive variables for the classification of (B) activation versus repression by both the canonical and non-canonical NF-κB pathway, and (C) regulation exclusively by the canonical versus exclusively by the non-canonical NF-κB pathway. (B and C): Combinations of NF-κB subunits are listed on the y-axis as binary vectors indicating the presence or absence of the subunits in the given order. Parameter estimates of the logistic regression models (x-axis) can be interpreted as log odds ratios and are indicated by circles. Confidence intervals (95%) are given by the vertical lines. Filled circles indicate that parameters are significantly different from zero (P<0.01). Missing estimates and confidence intervals occur when the specific subunit combination was not observed in the dataset. Bar plots on the right side indicate the percentage of bootstrap samples in which each parameter was significantly different from zero (P<0.01). The dashed line marks 90%.

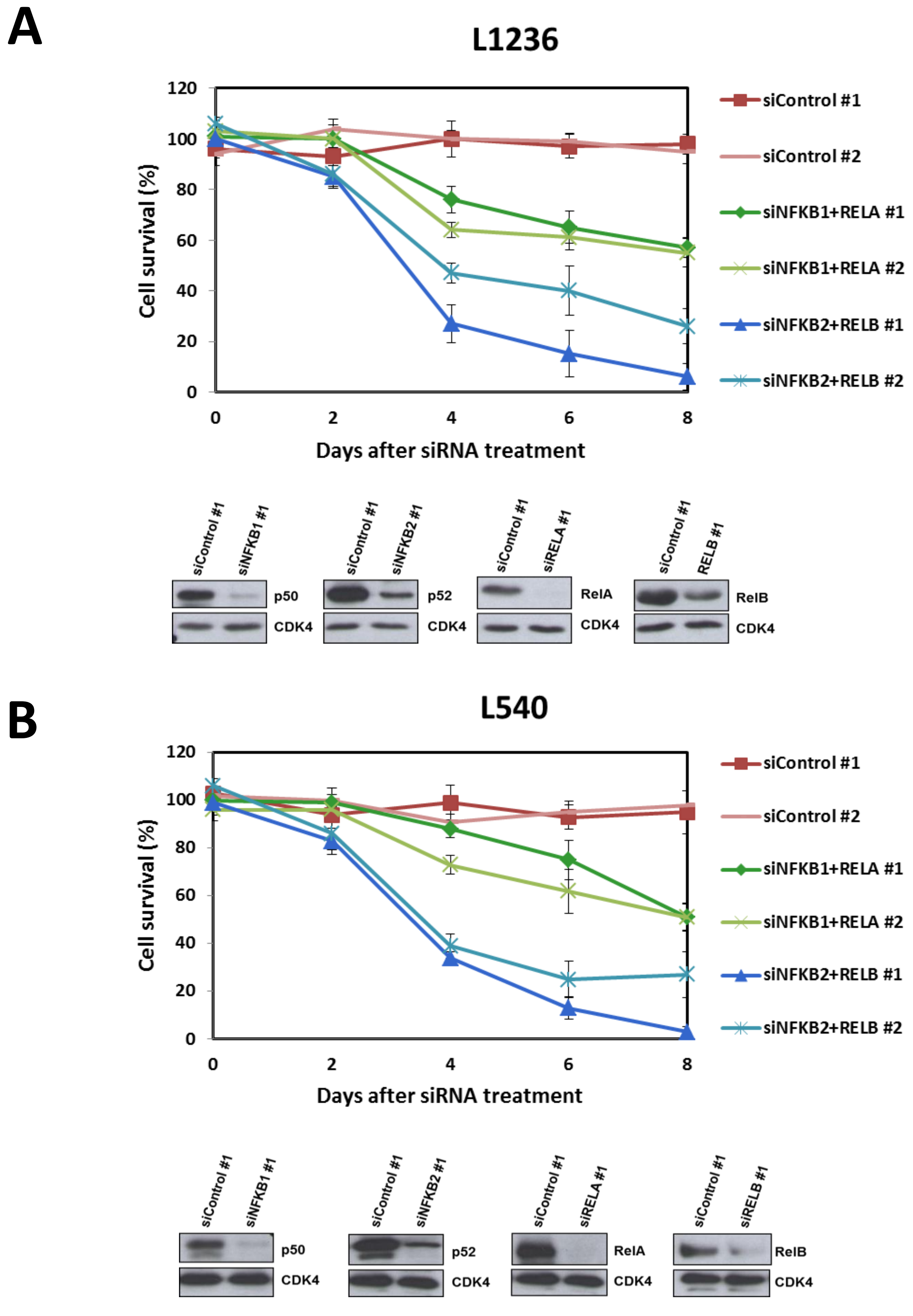

Figure S6 (related to Figure 6)

C

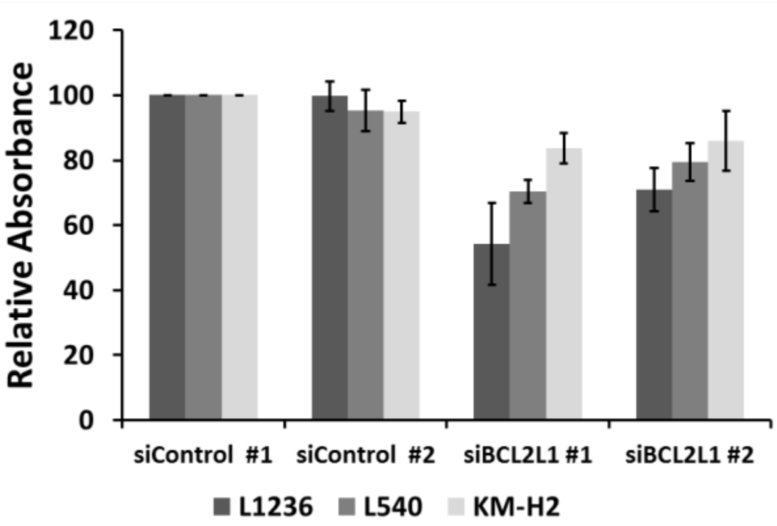

D

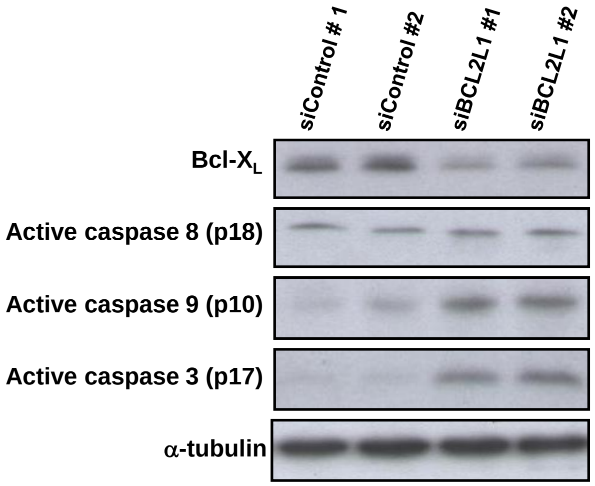

Continuation of Figure S6 (related to Figure 6)

Figure S6 (related to Figure 6). Depletion of p50/RelA and p52/RelB results in death of HL cells. Cells were incubated with siRNAs as indicated in the figure, and time-course experiments were performed to analyze cell viability. Two distinct siRNA sequences were used against each NF-κB subunit. Viability of the siRNA-treated cells was measured using Trypan blue and normalized by the number of viable cells in an untreated sample. (A) Top: Total number of viable L1236 cells relative to control. Bottom: KD efficiencies of siRNA sequences #1 visualized in samples collected 6 days after siRNA treatment are shown as representatives. (B) Top: Total number of viable L540 cells relative to control. Bottom: KD efficiencies of siRNA sequences #1 visualized in samples collected 6 days after siRNA treatment are shown as representatives. Cell viability was monitored with Alamar Blue assay. (C) HL cells (L1236, L540 and KM-H2) were incubated for three days with two siRNA sequences against BCL2L1. (D) WB showing BCL2L1 KD efficiency and expression of the activated forms of caspase 8 (p18), caspase 9 (p17) and caspase 3 (p10) in L-1236 cells. Curves represent the mean of the experimental values (n=3). Error bar = SEM of triplicate reads. CDK4 and α-tubulin antibodies were used as loading control.

A

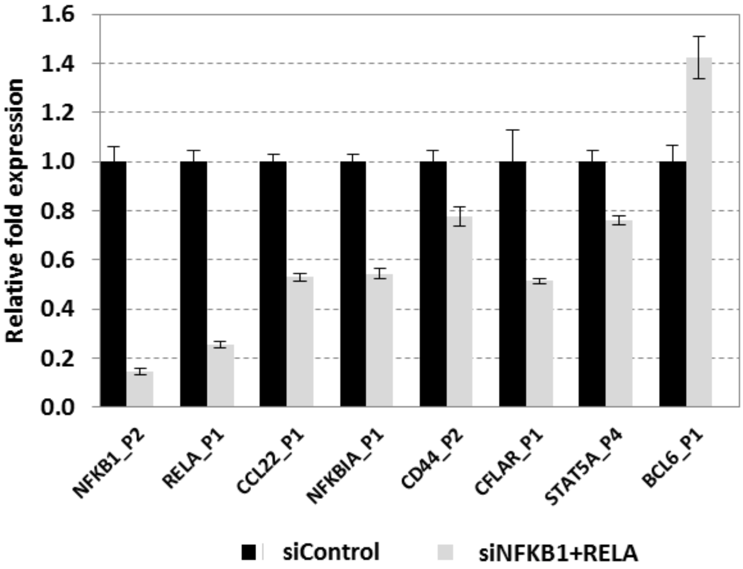

B

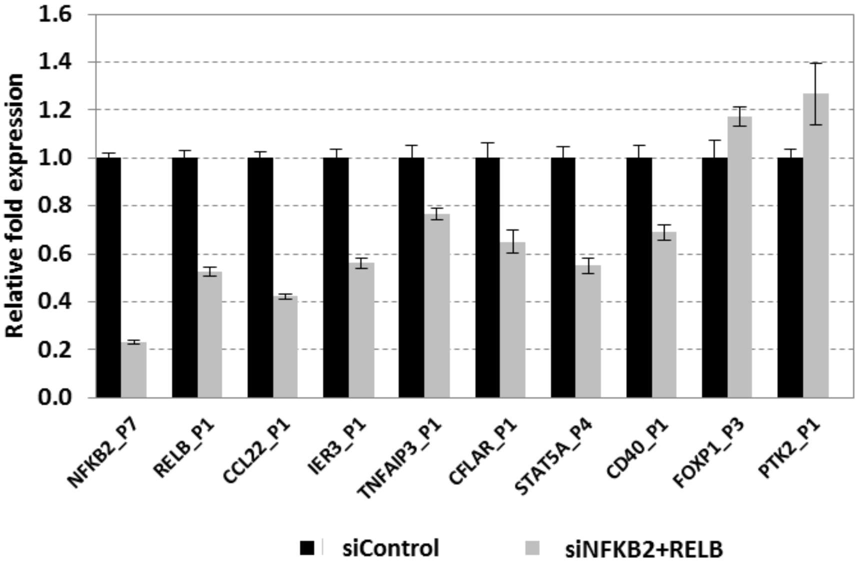

Figure S7 (related to Figure 7)

Figure S7 (related to Figure 7). Analysis of genes that were differentially expressed in classical HL compared to other malignant B cell malignancies and normal B cells. RT-qPCR determination of relative mRNA levels of up or down-regulated genes after KD of (A) p50/RelA or (B) p52/RelB in L1236 cells. Three housekeeping genes were used as references. Error bar = SEM of triplicate reads.
