# Supplementary material for: A roadmap of constitutive NF-κB activity in Hodgkin lymphoma: Dominant roles of p50 and p52 revealed by genome-wide analyses
Source: Genome Med. 2016 Mar 17;8:28. doi: 10.1186/s13073-016-0280-5 (PMC4794921; doi:10.1186/s13073-016-0280-5)
Supplement: Additional file 3: Table S1 — shows immunohistochemical analyses of HL biopsy samples with p100/p52 and p105/p50 antibodies. (DOCX 12kb) [file 13073_2016_280_MOESM3_ESM.docx]

**Additional File 3: Table S1**

| **Diagnosis** | **NF-κB subunit** | **Cases (n)** | **Score 3+** | **Score 2+** | **Score 1+** | **0** |
| --- | --- | --- | --- | --- | --- | --- |
| **cHL (total)** | p50 | 20 | 6 | 2 | 4 | 8 |
|  | p52 | 18 | 8 | 0 | 7 | 3 |
| **Subtype NS** | p50 | 9 | 3 | 2 | 2 | 2 |
|  | p52 | 8 | 3 | 0 | 3 | 2 |
| **Subtype MC** | p50 | 8 | 2 | 0 | 2 | 4 |
|  | p52 | 7 | 2 | 0 | 4 | 1 |
| **Subtype LP** | p50 | 3 | 1 | 0 | 0 | 2 |
|  | p52 | 3 | 3 | 0 | 0 | 0 |

**Table S1 (related to Figure 1)** Immunohistochemical analysis of lymph node sections of HL biopsy samples with p100/p52 and p105/p50 antibodies. A total of 60 % and 83 % of classical HL (cHL) samples showed positivity for nuclear p50 and p52, respectively. The heterogeneity of the nuclear staining was classified from strong (Score 3+) to weak (Score 1+). The following cHL subtypes were analyzed: NS (nodular sclerosis), MC (mixed cellularity), and LP (lymphocyte predominant).
