# Supplementary material for: A roadmap of constitutive NF-κB activity in Hodgkin lymphoma: Dominant roles of p50 and p52 revealed by genome-wide analyses
Source: Genome Med. 2016 Mar 17;8:28. doi: 10.1186/s13073-016-0280-5 (PMC4794921; doi:10.1186/s13073-016-0280-5)
Supplement: Additional file 6: Table S4 — shows the overlap of NF-κB subunit binding and DNAse I hypersensitive sites (DHS) in L1236 and GM12878 cells. (DOCX 13 kb) [file 13073_2016_280_MOESM6_ESM.docx]

**Additional File 6: Table S4**

| **Overlap^a^** | **Cell line^b^** | **RelA** | **RelB** | **p50** | **p52** | **any** |
| --- | --- | --- | --- | --- | --- | --- |
| ChIP | L1236  GM12878 | 492  19920 | 3984  16520 | 10975  4282 | 10893  10766 | 14865  27882 |
| ChIP in DHS | L1236  GM12878 | 0.81  0.68 | 0.65  0.60 | 0.48  0.73 | 0.51  0.79 | 0.46  0.65 |
| ChIP in any DHS | L1236  GM12878 | 0.97  0.71 | 0.87  0.63 | 0.77  0.76 | 0.75  0.81 | 0.74  0.67 |
| ChIP in ChIP | L1236  GM12878 | 0.74  0.02 | 0.66  0.18 | 0.26  0.68 | 0.34  0.38 | 0.54  0.33 |
| ChIP in DHS  ChIP in any DHS | L1236 specific | 0.87  0.91 | 0.66  0.79 | 0.49  0.73 | 0.48  0.67 | 0.43  0.60 |

**Table S4 (related to Figure 2)** Overlap of NF-κB subunit binding and DNAse I hypersensitive sites (DHS) in L1236 and GM12878 cells.

^a^ChIP: total number of ChIP regions; ChIP in ChIP: percentage of overlaping ChIP regions between L1236 and GM12878; ChIP in DHS: percent overlap of ChIP regions with DHS regions in the cell type indicated; ChIP in any DHS: percent overlap of ChIP regions with DHS regions in any of the two cell types.

^b^Percentage relative to the total number of subunit ChIP binding regions in the cell type indicated; ‘L1236 specific’ indicates binding exclusively in L1236 and not in GM12878.
