# Supplementary material for: A roadmap of constitutive NF-κB activity in Hodgkin lymphoma: Dominant roles of p50 and p52 revealed by genome-wide analyses
Source: Genome Med. 2016 Mar 17;8:28. doi: 10.1186/s13073-016-0280-5 (PMC4794921; doi:10.1186/s13073-016-0280-5)
Supplement: Additional file 8: Table S6 — shows the comparison of combinatorial subunit binding between L1236 and GM12878 cells. (DOCX 13 kb) [file 13073_2016_280_MOESM8_ESM.docx]

**Additional File 8: Table S6**

| **Binding pattern** | **GM12878** | | **L1236** | |
| --- | --- | --- | --- | --- |
| p50 p52 RelA RelB | **Total^a^** | **% overlap^b^** | **Total^a^** | **% overlap^b^** |
| 0001 | 3,510 | 0.77 | 570 | 4.21 |
| 0010 | 5,654 | 0.07 | 26 | 15.38 |
| 0011 | 6,891 | 0.00 | 44 | 0.00 |
| 0100 | 3,051 | 2.88 | 2,936 | 2.69 |
| 0101 | 319 | 0.94 | 287 | 1.39 |
| 0110 | 1,127 | 0.00 | 2 | 0.00 |
| 0111 | 3,048 | 0.10 | 25 | 12.00 |
| 1000 | 502 | 20.52 | 3,158 | 2.91 |
| 1001 | 38 | 0.00 | 151 | 0.00 |
| 1010 | 177 | 0.00 | 4 | 0.00 |
| 1011 | 344 | 0.00 | 19 | 0.00 |
| 1100 | 470 | 44.04 | 4,732 | 3.99 |
| 1101 | 72 | 25.00 | 2,539 | 0.28 |
| 1110 | 381 | 0.00 | 23 | 4.35 |
| 1111 | 2,298 | 8.79 | 349 | 56.45 |

**Table S6 (related to Figure** **2)** Comparison of combinatorial subunit binding between L1236 and GM12878 cells.

^a^Total number of regions with the given combinatorial binding pattern in the cell type indicated.

^b^Percentage of these regions that show an identical combinatorial pattern in the other cell type.
